# Dexmedetomidine for acute respiratory conditions requiring non-invasive respiratory support in pediatric and adult patients: systematic review and meta-analysis of the literature

**DOI:** 10.3389/fmed.2026.1740700

**Published:** 2026-04-24

**Authors:** Luisa Zupin, Lotte Wijers, Ludovica Barbi, Giorgio Cozzi, Francesca Foglio, Lorenzo Alvio De Luca, Filippo Pigani, Alessandro Amaddeo

**Affiliations:** 1 Pediatric Department, Institute for Maternal and Child Health IRCCS Burlo Garofolo, Trieste, Italy; 2 Emergency Department, Viecuri Medical Center, Venlo, Netherlands; 3 Pediatric Department, Maxima Medical Center, Veldhoven, Netherlands; 4 Department of Medicine, Surgery and Health Sciences, University of Trieste, Trieste, Italy

**Keywords:** acute respiratory distress, dexmedetomidine, non-invasive respiratory support, sedation, systematic review

## Abstract

**Background:**

Dexmedetomidine is increasingly used as an anxiolytic and sedative in pediatric patients with acute respiratory distress for managing anxiety and agitation. However, its effectiveness and safety in the pediatric population remain unclear, and clinical practice is often guided by evidence derived from adults.

**Aim:**

A systematic review was conducted to examine the evidence on the use of dexmedetomidine in patients undergoing non-invasive respiratory support (NRS) for acute respiratory conditions in both pediatric and adult individuals.

**Methods:**

A comprehensive literature search was conducted on PubMed, Web of Science and Embase up to September 2025, evaluating dexmedetomidine in patients requiring NRS. The risk of bias was assessed using JBI’s critical appraisal tools, and available comparative studies randomized controlled studies (RCT) were analyzed in a meta-analysis. Certainty was graded according to the GRADE (Grading of Recommendations, Assessment, Development, and Evaluations) methodology.

**Results:**

Ten studies evaluating dexmedetomidine in pediatric NRS were identified. While most studies suggested that dexmedetomidine may improve tolerance to NRS and reduce agitation, the pediatric evidence base consists mainly of observational studies without randomized trials, preventing a quantitative synthesis. Consequently, any direct comparisons with adult results cannot be directly extrapolated and should be considered strictly exploratory. Twenty-four studies (9 RCTs) were conducted in adult patients. The evidence from adult studies was more robust, showing that dexmedetomidine has the potential to reduce agitation, aid NRS acceptance, and decrease the need for intubation, as well as the incidence of delirium.

**Conclusion:**

Current evidence supporting the use of dexmedetomidine in pediatric patients undergoing NRS is promising but remains insufficient. Findings from adult populations suggest that dexmedetomidine can effectively reduce agitation and may facilitate NRS acceptance in various conditions requiring respiratory support. However, future robust randomized controlled trials in pediatric patients are needed to determine efficacy, optimal dosing and safety in children with acute respiratory distress. These data will enable the development of age-specific guidelines and recommendations, ensuring a safer and more effective use.

## 1 Introduction

Acute respiratory distress is a life-threatening condition characterized by hypoxemia or hypercapnia resulting from respiratory failure, often requiring respiratory support. Non-invasive respiratory support (NRS) has been increasingly used in the management of respiratory distress in both adults and children, providing a less invasive alternative to endotracheal intubation, which may be associated with complications. However, the effectiveness of NRS depends largely on patient comfort, tolerance and, when using non-invasive ventilation (NIV), synchronization with the ventilator, all of which can be negatively influenced by anxiety, agitation and dyspnea-related distress. Anxiety, fear, and pain have been shown to exacerbate tachypnea, leading to sharp breathing, breath holding, and further oxygen desaturation (1, 2).

Furthermore, in severe cases, in which NIV use might be required, agitation can further reduce mask tolerance, making NIV acceptance and maintenance more challenging, particularly in pediatric patients (3, 4). In these cases, sedative treatment is a pragmatic approach that can be used in both the emergency department and intensive care units. Treatment of agitation and distress in acute respiratory distress has been primarily based on benzodiazepines and opioids (5). Although effective, these agents can adversely affect hemodynamics and breathing drive. For these reasons, the use of dexmedetomidine in this context has been increasingly reported since its introduction in 1999. Indeed, dexmedetomidine, a selective alpha-2-adrenergic agonist, has gained considerable attention in critical care due to its sedative, anxiolytic and analgesic properties, along with minimal respiratory depression, preservation of breathing drive and limited mild hemodynamic effects. These properties stem from its pharmacological profile, selectively acting on alpha-2-adrenergic receptors in the locus coeruleus. Moreover, dexmedetomidine has the advantage of being administered via different routes, with intravenous administration being the most common. However, intranasal administration can also be particularly useful in an acute setting (6).

Despite its increasing off-label use in intensive care settings, the evidence for dexmedetomidine application in the emergency setting - especially in the pediatric population- remains limited and fragmented. Only limited evidence is available in the literature regarding its effectiveness as a supporting agent in acute respiratory distress requiring non-invasive ventilation, with some papers suggesting its effectiveness and others reporting no advantage, with notable differences in outcomes between children and adults (6–9). Clinical practice varies widely, and concerns persist about hemodynamic side effects and optimal dosing strategies. A clearer understanding of dexmedetomidine’s safety, efficacy and impact on NRS outcomes could inform clinical guidelines and enhance care for patients experiencing respiratory distress, ultimately reducing the need for intubation and improving morbidity and mortality.

This systematic review aims to synthesizing the current evidence on the use of dexmedetomidine for the management of acute respiratory distress requiring NRS in both children and adults. Specifically, it seeks to evaluate its effects on patient tolerance, intubation rates, sedation quality, respiratory parameters and adverse events. By consolidating existing data, this review aims to clarify the clinical role of dexmedetomidine in this context and identify knowledge gaps warranting further research.

Although the included respiratory conditions have diverse pathophysiologies, they share a common clinical challenge: anxiety, agitation, and interface intolerance often precipitate in non-invasive support failure. Therefore, we included these heterogeneous conditions to pragmatically evaluate dexmedetomidine’s role in targeting this shared pathway—facilitating patient comfort and ventilator synchrony—rather than treating specific disease mechanisms.

## 2 Methods

### 2.1 Literature search

The systematic review adhered to the PRISMA (Preferred Reporting Items for Systematic Reviews and Meta-Analyses) guidelines (10).

The study was registered in PROSPERO (n. CRD420251089195).

### 2.2 Search strategy

Three authors (L.Z., L.W, L.B.) conducted the manual literature search on PubMed,^1^ Web of Science^2^ and Embase^3^ databases up to September 2025, using as key terms, “non-invasive ventilation,” “CPAP,” “high flow cannula,” “dyspnea,” and “acute respiratory distress,” “acute respiratory failure,” “bronchiolitis,” “asthma” and “dexmedetomidine.” Moreover, the ClinicalTrials.gov (for clinical trials) registry and ProQuest (for dissertations and theses) were examined. The full search strategy was reported in Supplementary Table 1.

### 2.3 Articles selection

Potential study titles and abstracts were manually screened for relevance, followed by full-text evaluation. Studies were independently screened for inclusion by three authors. Duplicates across databases were manually merged. Studies conducted in both the emergency department and the intensive care unit (ICU) were included to capture all indirect evidence.

Inclusion criteria:

- Clinical studies on human subjects
- Pediatric or adult patients
- Use of dexmedetomidine in the context of non-invasive respiratory support, including NIV, Continuous Positive Airway Pressure (CPAP), and high flow nasal cannula in the presence of respiratory conditions, including dyspnea, acute respiratory distress, acute respiratory failure, bronchiolitis, and asthma.
- Articles in the English language

Exclusion criteria:

- Use of invasive ventilation
- Palliative or procedural setting (endoscopy or surgery)
- Non-respiratory conditions treated with dexmedetomidine
- pre-clinical *in vivo*, *in vitro*, or pharmacological studies
- articles not in the English language

The discrepancy between the authors regarding study selection was resolved by involving another author.

### 2.4 Risk of bias assessment

Five authors independently assessed the risk of bias using the Joanna Briggs Institute (JBI)’s critical appraisal tools, tailored to various study designs, including intervention studies, observational cohorts, and case series (11–13). Each checklist item was rated as “Yes,” “No,” “Unclear,” or “Not applicable” in accordance with the official guidelines. Discrepancies were resolved by consensus between authors. The results were visualized using the “robvis” online tool (14).

### 2.5 Data extraction

The data were manually extracted from the articles by an author (L.Z.), whereas other authors (L.W, L.B. F.P, L.A.D.) checked the correctness of the data, specifically, age, sex, medical condition, and sedation treatment were recorded, as well as outcomes, such as intubation rate, mortality rate, incidence of delirium, NRS duration, and length of stay (LOS) in intensive care unit (ICU).

Intubation rate was defined as the proportion of patients who failed NRS treatment and required intubation, and mortality was defined as the proportion of patients who died. Incidence of delirium indicated the number of delirium cases occurring during NRS therapy. NRS duration referred to the length of time patients received NRS, while LOS in ICU represented the total duration of hospitalization in the ICU.

### 2.6 Meta-analysis

Only randomized controlled studies (RCT) studies that compared the use of dexmedetomidine to facilitate NRS acceptance to another drug or a placebo were included in the meta-analysis.

The primary outcome was the incidence of intubation, while the mortality rate, incidence of delirium, duration of NRS, and LOS in ICU were considered secondary.

The statistical analysis was performed in R Studio (15, 16) with the “meta” package (17).

For dichotomous outcomes (incidence of intubation, mortality rate, and incidence of delirium), pooled risk ratios (RRs) with 95% confidence intervals were calculated using random-effects models (REM). For continuous outcomes (duration of NRS and LOS in ICU), inverse variance weighting was employed to compute mean differences (MD). Heterogeneity across studies was assessed using the *I*^2^ statistic and *τ*^2^. Influence diagnostics were conducted to identify outlier studies that may disproportionately affect the pooled estimates.

The Grade of Evidence was assessed according to the guidelines of the Grading of Recommendations, Assessment, Development, and Evaluations (GRADE) framework (18). Specifically, GRADE was used to ensure confidence in the evidence and the strengths (or weaknesses) of our recommendations. The rating was based on the risk of bias (assessed by JBI’s critical appraisal tools), effect estimates (derived from the meta-analysis using REM), inconsistency (variation of the results across the studies, as measured by *I*^2^), indirectness (determining if the evidence directly addressed the research question), imprecision (degree of uncertainty evaluated by 95% CI), publication bias. The final rating was determined by consensus among all the authors.

## 3 Results

**Figure 1:**
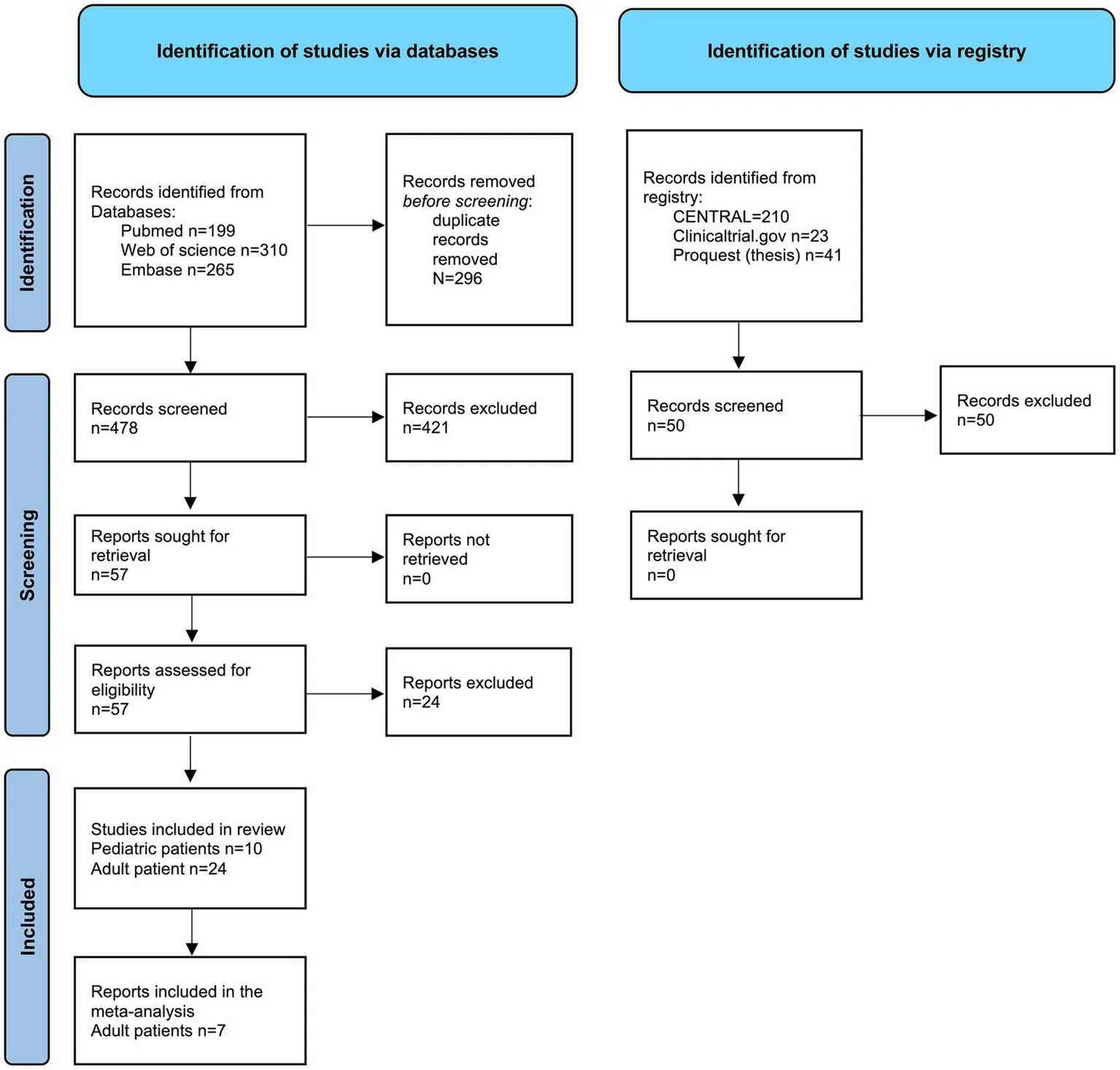
Flowchart of the study.

The flowchart of the literature search strategy is shown in Figure 1. A total of 199 studies were initially identified in PubMed, 310 in Web of Science and 265 in Embase. After removing 296 duplicates, 478 articles (title and abstract) were screened for eligibility. Moreover, CENTRAL, clinicaltrial.gov and Proquest were searched (*n* = 274), but no additional studies were identified. Overall, 57 articles were retrieved for full-text assessment. Finally, 33 articles were included in the review, 10 conducted in pediatric patients and 24 in adults (Tables 1, 2).

**Table 1:** Characteristics of studies conducted on pediatric patients.

Study (reference)	Study type	Patients (age, sex)	Clinical condition	Intervention	Outcomes	Key results	Adverse effect if any
Observational studies							
Bermúdez-Barrezueta (19)	Prospective, multicenter, observational trial	457 patients (median age 3.3 months, IQR 1.3–16.1) M 54.7%, F 45.3%	Children with acute respiratory failure (ARF) Bronchiolitis Bronchospasm Pneumonia Cardiogenic pulmonary edema Sepsis	NIV 213 (46.6%) children required sedation with various sedative agents Dexmedetomidine used in 23.5% of cases (median initial dose of 0.5 μg/kg/h and median cumulative dose of 16.5 μg/kg)	NIV success in 95.6% of patients, intubation 6.1% in sedation group and 2.9% in non sedation group	Sedation can help in improving the physiological parameters and comfort status during NIV of children < 5 years with ARF	Adverse effects associated with sedations were recorded in 8% of patients: 11 cases of bradycardia in those who received dexmedetomidine (*n* = 33), 2 cases of bradycardia and 1 case of respiratory depression in those who received benzodiazepines (*n* = 102), 1 case of respiratory depression in a patient who received propofol (*n* = 13)
Cousin (26)	Retrospective study	49 patients. Median age was 61 days (IQR 44–73) sex NR	Severe bronchiolitis failing initial NIV support	Non-invasive neurally adjusted ventilatory assist ventilation (NIV-NAVA) with a total face mask interface (TFM) IV dexmedetomidine (0.3–1 mcg/kg/h)	No patients required intubation and no patient experienced intolerance	Respiratory support with TFM-NIV-NAVA is a feasible rescue therapy in bronchiolitis	Not reported
Christian (20)	Retrospective study	43 patients 14 no delirium median 0, (0–10), 79% F, 21% M, 29 delirium 1, (0–17). F 48%, M 52%	Pediatric ICU patients with respiratory insufficiency (viral bronchiolitis, pneumonia, influenza A infection, and status asthmaticus.) requiring NIV.	NIV: BiPAP-bilevel positive airway pressure; 26, CPAP 2, high-flow nasal cannula- HFNC 27 Various drugs among them dexmedetomidine	All patients weaned off NIV and experienced NIV discontinuation delirium in 29 of 43 patients.	Delirium is common in patients requiring NIV due to sedative agent, (mainly benzodiazepine).	Delirium was observed in 28 of the 36 patients who received dexmedetomidine; 19 of these patients also received benzodiazepines
Eidman (6)	Retrospective cohort study	137 no sedation (mean age 96 months range 3–237), F 47%, M 53%; 68 sedation (40 months 3–193), F 46%, M 54%	Asthma, Bronchiolitis, Pneumonia, viral respiratory infection, Aspiration pneumonia, Chronic lung disease exacerbation	NIV dexmedetomidine (range 0.2–1 mg/kg/h)	Dexmedetomidine patients experienced an intubation rate of 13.09% while no sedated patients of 12.41%	Dexmedetomidine may improve NIV tolerance in patients with acute respiratory failure without increasing risk for intubation	In the sedation group (*n* = 68) 10 patients developed bradycardia, which was transient and improved spontaneously, and 8 patients experienced hypotension, although the authors attributed it to other causes
Shutes (23)	Retrospective chart review	0–18, (1–24 months 76%) F 41%, M 59%	382 PICU patients Bronchiolitis Asthma Pneumonia Viral pneumonia Aspiration pneumonia	Positive Pressure ventilation dexmedetomidine infusion (starting dose 0.4 μg/kg/h range 0.4–0.5, median peak dose of 1 μg/kg/h range 0.6–1.2)	336 patients (88%) experienced dexmedetomidine discontinuation, and 19 patients (5%) withdrawal.	Dexmedetomidine use for noninvasive positive pressure ventilation sedation presented hemodynamic alterations. Withdrawal was associated with higher doses	The hemodynamic alterations observed 4 h after reaching the peak dose included bradycardia in 28% of patients, hypertension in 33%, and hypotension in 2%; during the dose escalation phase, bradycardia was observed in 75% of patients and hypotension in 30%
Venkatraman (24)	Observational cohort study.	202 patients Age, median (IQR) 2.0 (1.0–4.0), F 52%, M 48%	Status asthmaticus, Acute bronchiolitis oncologic diagnosis Croup Septic shock	NIV CPAP, BiPAP, or high-flow nasal cannula Dexmedetomidine infusion (0.61 μg/kg/h) (range 0.4–0.8)	168 patients (83%) achieved target sedation level for at least 80% of the NIV duration. 96% the outcome of NIV was successful	Dexmedetomidine was effective as continuous sedative infusion during NIV	Bradycardia was observed in 26 patients (13%), hypotension in 41 patients (20%), and hypopnea in 11 patients (5%). Endotracheal intubation was required in 5 patients (2.5%), including 1 patient (0.5%) who experienced cardiorespiratory arrest.
Case series							
Musolino (25)	Retrospective, observational, consecutive case series	26 patients 72 months median range (16–345), F 42%, M 58%	Moderate-to severe bronchiolitis	Helmet Continuous Positive Airway Pressure one-time bolus dose of dexmedetomidine (0.25 mcg/kg/dose)	Pharmacological sedation with a single dose of dexmedetomidine was required in 15/26 patients (57%) 22 patients tolerated the interface	In non-intensive settings H-CPAP for infants with bronchiolitis may be feasible	2 patients developed relative bradycardia
Piastra (22)	Retrospective case series	40 patients Median age was 16 months (IQR 6,5; 33.50), F 25% M 75%	Acute respiratory failure Bronchiolitis ARDS Chest trauma Burn-associated Respiratory Failure Status Asthmaticus Neurological illness Pneumonia Bronchopulmonary Dysplasia Post-operative patients	NIV Helmet 12, Total Face Mask 11, Nasal Mask 17 dexmedetomidine IV (start 0.5–0.7 mcg/kg/h until 1.0–1.4 mcg/kg/h)	No NIV discontinuation due to NIV intolerance	Dexmedetomidine should be considered safe and help in improving NIV and reduced+G8 agitation status	The authors report a significant decrease in heart rate and mean arterial pressure 2 h after the introduction of dexmedetomidine; however, no patient developed severe bradycardia or hypotension requiring interruption of the infusion or administration of rescue medications.
Case report							
Cozzi 2017 (9)	Case report	2 patients 15 months (F) and 4 years old (F)	2 patients with acute asthma exacerbation	Oxygen supplementation with high-flow nasal cannula albuterol IV dexmedetomidine intranasal at 3 mcg/kg	Oxygen saturation from 88 to 93%, 90–94%	Dexmedetomidine allowed to rest and improved tolerance to therapy	Not reported
Lichtsinn (21)	Case report	2 pediatric patients 8 week old (M), 27 month old (M)	Acute hypoxemic respiratory failure due to RSV bronchiolitis, pulmonary hypertension secondary to pulmonary hypoplasia	BiPAP dexmedetomidine infusion was started at 0.2 mcg/kg/h up to 0.7 endotracheal intubation 0.3 mcg/kg/h 1.1 mcg/kg/h, up to 1.2	Dexmedetomidine discontinuation	Dexmedetomidine increased vagal tone episodes resulting in asystole	In the first case, the patient experienced bradycardia with episodes of asystole lasting up to 6 s In the second case, the patient exhibited a 15-s period of asystole requiring cardiopulmonary resuscitation, followed by bradycardia

ARDS, Acute Respiratory Distress Syndrome; BiPAP, Bilevel Positive Airway Pressure; CPAP, Continuous Positive Airway Pressure; H-CPAP, Helmet Continuous Positive Airway Pressure; HFNC, High-Flow Nasal Cannula; IQR, Interquartile Range; IV, Intravenous; NIV, Non-Invasive Ventilation; NIV-NAVA, Non-Invasive Neurally Adjusted Ventilatory Assist; PICU, Pediatric Intensive Care Unit; RF, Acute Respiratory Failure; RSV, Respiratory Syncytial Virus; TFM, Total Face Mask (ventilation interface).

**Table 2:** Characteristics of studies conducted on adult patients.

Study (reference)	Study type	Patients (age, sex)	Clinical condition	Intervention	Outcomes	Key results	Adverse effect if any
Observational studies/RCTs							
Abdelgalel (28)	Randomized, double blinded controlled study	90 patients 30 dexmedetomidine mean 51.1 ± 8.4 (F 2 0%, M 80%) 30 haloperidol mean 51.0 ± 8.8 SD (M 73%, F 27%) 30 placebo mean 49.1 ± 8.0 SD (M 70%, F 30%)	Acute exacerbation of acute respiratory failure in COPD, acute hypoxemic cardiogenic pulmonary edema, postoperative respiratory failure patients	NIV dexmedetomidine loading dose of 1.0 lg/kg, then 0.2–0.7 lg/kg/h haloperidol loading dose 2.5 mg, then, 0.5–2 mg/h placebo group saline infusion	Incidence of delirium, NIV duration, length of ICU and in hospital, were reduced in dexmedetomidine treated patients respect to haloperidol and placebo groups Mortality rate was inferior in dexmedetomidine, and haloperidol patients respect to placebo.	Dexmedetomidine is more effective in preventing delirium during NIV compared to haloperidol. Furthermore, there was a lower incidence of endotracheal intubation, NIV failure and shorter stay on ICU in patients that received dexmedetomidine compared to patients that received haloperidol.	Bradycardia: dexmedetomidine group n.8, haloperidol n.2, placebo n.1. prolonged QTc-interval (>500 ms): haloperidol n.2 arrhythmia: dexmedetomidine group n.2, haloperidol n.3, placebo n.2 Hypotension: dexmedetomidine group n.4, haloperidol n.3, placebo n.3 vomiting: dexmedetomidine group n.3, haloperidol n.5, placebo n.3.
Altınkaya Çavuş (33)	Prospective randomized cohort study	227 patients dexmedetomidine low L dose median 66 (IQR 60–82) F 61%, M 39% dexmedetomidine high H dose 74 (59–80) F 39%, M 61% remifentanil low dose 71 (67–77) F 61% M39% remifentanil high dose 72 (62–76) F 26%, M 74% propofol PROP 71.5 (64.5–76) F 36%, M 64%	NIV intolerance, acute respiratory acidosis, a diagnosis of COPD, and respiratory distress	NIV dexmedetomidine IV loading 1 μg/kg, then 0.2 μg/kg/h in the DEXL group and at 0.6 μg/kg/h in the DEXH group. Remifentanil: initial at 1 μg/kg, then 0.03 μg/kg/h in the REML group, and at 0.06 μg/kg/h in the REMH group. propofol: IV initial at 0.3 mg/kg/h, then, any increases and/or decreases 0.1 mg/kg/h.	NIV failure: 2 (8.7%) DEXL, 3 (13%) DEXH, 7 (30.4%) REML, 13 (56.5%) REMH, and 20 (71.4%) PROP Mortality: 1 (4.3) DEXL, 2 (8.7) DEXH, 5 (21.7) REML, 5 (21.7) REMH, 10 (35.7) PROP NIV duration, ICU and hospital length of stay were lower in DEXH and DEXL groups	NIV failure, mortality, ICU LOS, IMV time, and hospital LOS were found to be lower with dexmedetomidine.	Side effects: 1 DEXL, 3 DEXH, 1 REML, 8 REMH, 8 PROP Apnea: 0 in DEXL; DEXH; REML, 1 REMH, 7 PROP higher incidence of bradycardia in the DEXL (4.3%) and DEXH (8.7%) groups than in the other groups (0%), (not statistically Significant) Not other side effects
Basilim (37)	Multicenter, retrospective cohort study	291 patients mean age SD 59.6 (15.46) M 61.4%, F 38.6% 259 control 32 dexmedetomidine	COVID-19 in the ICUs	Noninvasive mechanical ventilation dexmedetomidine does not reported	NIV failure is higher in control group Time to invasive mechanical ventilation was longer in control. Longer ICU LOS but shorter hospital LOS ICU length of stay in dexmedetomidine group. However, all these comparison were not significantly different between the 2 groups. In hospital mortality was similar	Dexmedetomidine did not lower the risk of respiratory failure requiring invasive ventilation. Though the mean time on invasive ventilation was lower in the dexmedetomidine group.	No significant difference in ICU related complications between both group such as kidney failure, shock, liver injury or delirium.
Deletombe (29)	RCT	19 patients with chest trauma Age median 56 (IQR 29, 67), F 11%, M 89%	Patients with Chest trauma who needed NIV	Infusion of DEX (0.7 mcg/kg/h) vs. placebo to improve NIV tolerance	Dexmedetomidine prolonged the duration of NIV compared to placebo: 280 min versus 120 min Dex associated to lower score for RASS and respiratory discomfort	Dexmedetomidine could facilitate the acceptance of the first session of non-invasive ventilation for patients with chest trauma	Bradycardia 1 arterial hypotension 5
Devlin (8)	RCT	Adults, 16 received DEX, 17 received placebo, dexmedetomidine mean age 68 (SD 6), F 31% M 69% Placebo mean age 62 (SD 17), F 65% M 35%	ARF and NIV	Randomization: IV dexmedetomidine (0.2 m g/kg/h and then titrated by 0.1 m g/kg/h every 30 min) or placebo up to 72 h, until NIV was stopped for 2 h, or until intubation	Use of dexmedetomidine did not impact on NIV tolerance, sedation level, intubation rate or duration of invasive ventilation. Dex incremented NIV duration	Dexmedetomidine did not improve NIV tolerance nor time tolerating NIV or remaining at the desired level of sedation	Severe bradycardia or hypotension did not develop in any patient in either group
Dumbar (39)	Observational multicenter cohort retrospective	433,357 patients on NIV who received sedation or analgesia, mean age 67 (SD 14) F 52%, M 48%	Patients with ARF and NIV	26.7/f the patients received sedation or analgesia. 11%received opioids only, 9% benzodiazepines only, 5% opioids and benzodiazepines, 0.4% dexmedetomidine only, 0.6% dexmedetomidine + opioids/benzodiazepines	Exposure to medications was associated with significantly increased odds of need for invasive ventilation or mortality use of dexmedetomidine was associated with greatest risk of intubation or mortality.	The use of sedation during NIV for ARF is potentially harmful	No reported
Ghazaly (35)	RCT	45 patients, 15 dexmedetomidine, 15 ketamin3, 15 placebo, age median 36 (IQR 24.5–47.5) M 95.6%, F 4.4%	Blunt chest trauma	Dexmedetomidine infusions initial 0.7 μg/kg/h, up to a maximum dose of 1.3 μg/kg/h. Ketamine infusion initial 0.20 mg/kg/h, up to a maximum dose of 0.5 mg/kg/h	NIV duration longer in patients receiving dexmedetomidine or ketamine respect to placebo RASS lower in dexmedetomidine group than ketamine or placebo	The use of dexmedetomidine improved NIV tolerance	Not reported
Hao (30)	RCT	179 patients, 89 remifentanil, 90 dexmedetomidine age 63 years (56–70 IQR), F 31% M 69%	NIV and ARF in cardiac surgery patients	Remifentanil IV an initial dosage of 0.05 μg/kg/min, dexmedetomidine an initial dosage of 0.5 μg/kg/min increment of 0.01 μg/kg/min for remifentanil and 0.1 μg/kg/h for DEX.	Initially the mitigation of NIV intolerance was better when using remifentanil, as well as the cumulative effect over time, however after 6 h the mitigation rate was similar. No differences between the two groups for NIV failure, reintubation, tracheostomy, ICU LOS, and mortality	No differences between remifentanil ad dexmedetomidine to achieve mitigation	Remifentanil group, no adverse effect dexmedetomidine 3 bradycardia and 6 severe hypotension
Huang (34)	RCT	62 patients (29 treated with midazolam, 33 with DEX) Midazolam, age 61.5 ± 7.3, F 59%, M 41% dexmedetomidine age 67.4 ± 8.2, F 58%, M 42%	Acute pulmonary edema and hypoxemia in NIV failure due to discomfort	Loading dose up to 1 μg/kg dexmedetomidine or 0.05 mg/kg midazolam. Maintenance infusion dose 0.2–0.7 μg/kg/h dexmedetomidine and 0.05–0.1 mg/kg/h midazolam.	Sedative scores in both groups. NIV failure was lower when using dexmedetomidine. Duration of mechanical ventilation and the ICU duration was decrease in dexmedetomidine group.	Dexmedetomidine performed better than midazolam in achieving sedation and awaking sedation, it decreased duration of mechanical ventilation and the ICU LOS	Bradycardia without medical intervention dexmedetomidine-18.2% midazolam 0%. Hypotension 12.1% (mid 17) Hypotension with intervention 3%(mid 3.4%) Delirium 3% (mid 13) Vomit 6.1% (mid 31) Gastric aspiration 6% (mid 10) Respiratory infections 9% (mid31)
Kim (40)	Observational prospective multi-center	155 patients treated with NIV, 26 received sedation 71.7 ± 11.4, F 34.6%, M 65.4% 129 control no sedation age 71.7 ± 11.5 years, F 39.5%, M 60.5%	ARF due mainly to exacerbation of COPD or pneumonia	26 patients who received analgo-sedation (15 remifentanil, 8dex, 1 fentanyl, 1 midazolam, 1 morphine)	Sedation group experienced higher NIV failure and intubation mainly due to inadequate efficacy and less to discomfort (13–14%). LOS was similar in the two groups as well as mortality rate	Sedation can be used for pain control in some patients and may be not unsafe	Sedation group 9 complications mainly skin erythema control group 45 complication, mainly skin erythema, large leak
Matsumoto (41)	Observation cohort	120 patients analyzed. 83 received sedation, adults	Acute respiratory distress syndrome/acute lung injury/severe pneumonia or acute exacerbation of interstitial pneumonia.	Sedation (risperidone, haloperidol, dexmedetomidine, midazolam, propofol, morphine, fentanyl) was performed only intermittently in 72 (60%) patients, was switched to continuously in 37 (31%) and was applied only continuously in 11 (9%) dexmedetomidine IV initial dose 0.2 μg/kg/h, increasing dose 0.1 μg/kg/h	In group non DNI (do not intubate), mortality and intubation rate did not differ between continuous or intermittent sedation. Among the DNI patients’ mortality higher in continuous use.	Sedation may be used at avoid NIV failure in agitated patients, however, a continuous use may lead to increased hypercapnic state and the possibility of increased mortality.	Midazolam 1 hypotension 1 delirium, 1 ileus, 3 hypercapnia pre-sedation progress and exhibited drowsiness Not side effects of DEX reported
Riccardi (42)	Observational retrospective	66 patients who underwent sedation for NIV Ketamine-dexmedetomidine 78.33 ± 8.72, F 59.1%, M 40.9%, ketamine 79.41 ± 10.76, F 59.1%, M 40.9%, dexmedetomidine 77.21 ± 12.33, F 55.5%, M 44.5%	Acute respiratory distress due to COVID19 pneumonia	NIV CPAP 22 patients ketamine (1 mg/kg, followed by 1 mg/kg/h) and dexmedetomidine (0.7 mcg/kg, followed by 0.7 mcg/kg/h). 22 patients DEX (0.7 mcg/kg in 10 min, followed by 0.7 mcg/kg/h up to 2 mcg/kg/h) 22 patients ketamine (1 mg/kg, followed by 1 mg/kg/h)	The combination of ketamine- dexmedetomidine resulted in faster sedation rates and better hemodynamic profiles than dexmedetomidine alone	The combination of ketamine- dexmedetomidine is beneficial and effective for NIV adherence particularly in ICU	Dexmedetomidine: 10 hypotension ketamine: 10 hypotension ketamine dexmedetomidine: Hypotension in 10 out of 12 patients treated with DEX only
Senoglu (32)	RCT	40 patients in NIV dexmedetomidine median 58 (range 28–80) F 55%, M 45% midazolam median 60 (range 30–81), F 50%, M 50%	Acute respiratory failure due to acute exacerbations or COPD	NIV dexmedetomidine: loading dose 1 μg/kg IV r 0.05, maintenance i0.5 μg/kg/h midazolam: loading dose 0.05 mg/kg, maintenance 0.1 mg/kg/h	Low level of RSS at 4 h in dexmedetomidine group respect to midazolam High level of RSAS at 8 h in dexmedetomidine group respect to midazolam high BIS in dexmedetomidine group respect to midazolam.	Similar effectiveness for Dexmedetomidine and midazolam in NIV patients. Dexmedetomidine respect to midazolam required fewer dose adjustments	No patient experienced adverse cardiovascular effects midazolam 1 oversedation 2 excluded for agitation
Simioli (43)	Retrospective cohort study	170 patients median age 71 (IQR 61–80), F 29%, M 71%	Interstitial pneumonia with critical extension compatible with severe COVID-19, acute respiratory failure and moderate ARDS	Non-invasive ventilation (NIV) and high-flow nasal cannula (HFNC). dexmedetomidine initial dose of 0.3 mcg/kg/h, maintenance dose between 0.2 and 0.8 mcg/kg/h	Increased pO2/FiO2 ratio. Reduction of NIV duration and reduction of endotracheal intubation in DEX group compared to the controls.	The adjunctive therapy with dexmedetomidine is associated with a higher pO2/FiO2, lower duration of NIV, and a lower risk of NIV failure	A higher incidence of sinus bradycardia was observed in the DEX group Hypotension and delirium as controls Bradycardia 30% (vs 18% controls)
Sinnot (44)	Cohort retrospective	103 patients in emergency department, age median 54 (IQR 37–65), F 32.2%, M 67.8%	ARF, dexmedetomidine for mechanical ventilation and NIV facilitation	0.4 micrograms/kilogram/h (mcg/kg/h) in non-intubated patients. Mechanically ventilated patients starting dose 0.7 mcg/kg/h, titration of 0.1 mcg/kg/h every 45 min	No differences was observed between the groups with/without adverse effects for ventilator-free days, ICU-free days, and hospital-free days and mortality. Duration of DEX treatment in the ED correlated with increased risk of hypotension or bradycardia	While adverse events are relatively common, they are of questionable clinical significance.	Hypotension (39%) bradycardia <60 bpm (17.5%). Dexmedetomidine interruption due to adverse event (7.8%) Non bradycardia under 40 bpm 18% of hypotensions were over 30% from baseline
Ueno (31)	RCT	24 patients 12 dexmedetomidine 12 not treated median age 76 (IQR 70, 78) F 37.5%, M 62.5%	Critical ill patients with ARF, at admission diagnosis was cardiovascular surgery, Acute respiratory failure, congestive heart failure, acute myocardial infarction	High-flow nasal cannula oxygen therapy (HFNC) dexmedetomidine initially at 0.4 μg / kg / h, then 0.2 to 0.7 μg / kg / h	Increment of Total Sleep Time and sleep efficiency in patients treated with dexmedetomidine	Dexmedetomidine improved sleep quantity without any side events in critically ill patients	Similar frequency of respiratory depression and hemodynamic instability in the two groups hypotension 1/12 in dex, 0/12 in non dex; bradycardia 0/12 For each group
Yildirim (45)	Retrospective cohort study	60 patients mean age 68 ± 11 SD, F 30% M 70%	Patients underwent major abdominal surgery gastrectomy, colorectal surgery, liver surgery, and pancreatectomy	Dexmedetomidine IV loading dose of 0.2 mcg/kg for 10 min, maintained at 0.2–0.7 mcg/kg/h	RASS score improved 92.5% of patients. Achieved the targeted sedation level. 7 patients experienced NIV failure and required intubation for worsening 56 patients improved and were discharged from ICU	Dexmedetomidine demonstrates effective sedation in patients with postoperative ARF during NIMV application after abdominal surgery. Dexmedetomidine can be considered safe and capable of improving NIMV success.	6 bradycardia and 5 hypotension 10%bradycardia (No one Needed intervention); 8.3% hypotension, (No one Needed intervention)
Baumgartner (38)	Prospective observational cohort study	75 patients, median age 62 (IQR 47.5–67), F 43%, M 57%	Single-center observational cohort study of patients treated with intravenous DEX for any indication in the ED.	DEX was administered in the ED for a median of 2.6 h (interquartile range [IQR] 1.6–4.9 h), with a median infusion rate of 0.3 μg/kg/h	Clinicians felt DEX was highly effective (median [IQR] effectiveness score of 5 [3–5] on a 5- point Likert scale). The median (IQR) ED Richmond Agitation Sedation Scale post- DEX was −1 (−4 to 0)	ED clinicians have a positive perception of the effectiveness of DEX	Any HAE (hemodynamic adverse effect), in 22 patients (29%), including hypotension in 13 (17%) and bradycardia in 10 (13%). Clinically significant HAE (Hemodynamic adverse events (HAEs)) in nine patients (12%), (4%). No patient required CPR, ECMO, or cardiac pacing
Case reports							
Akada (36)	Prospective clinical investigation	10 patients mean age 71, range 46–80, F 30% M 70%	Acute respiratory failure, dyspnea of sudden onset, typical findings on chest radiograph, hypoxemia- acute respiratory distress	NIV dexmedetomidine loading dose of 1.0 lg/kg, then 1continuous infusion at a dosage range of 0.2 to 0.7 microg/kg or by continuous infusion at a dosage of 0.7 microgram/kg. Haloperidol loading dose 2.5 mg, then, 0.5–2 mg/h placebo group saline infusion	The primary outcome was agitation measured with the Ramsay and RASS score. Effective sedation was obtained in all sedated patients. The Pao2/Fio2 ratio and Paco2 and respiratory function improved. no intubation, all patients weaned from NIV and were discharged from ICU alive	Dexmedetomidine was effective medication to reduce agitation in these patients.	Not reported
Akhtar (46)	Case series	6 patients 51, 68, 40, 50, 54 years old, 4 M, 2 F	Acute respiratory distress, 5 COVID-19, 1 acute exacerbation of COPD.	NIV dexmedetomidine IV initial 0.2 to 0.3 mcg/kg 10–15′, then 0.3–0.4 mcg/kg/h	The primary outcome was difference in agitation after dexmedetomidine measured with RASS score. The patients had RASS of +3 before the intervention, and −1 or −2 after dexmedetomidine. Moreover, oxygenation improved in 4/6 patients.	Dexmedetomidine was found to an effective sedative for NIV in this patient population	No adverse effect
Demuro (47)	Case report	91 year old female	In ICU for COPD and Heart failure exacerbations in pneumonia	Patient in BIPAP, infusion of DEX (starting 0.2 μg/kg/h, and continued on doses ranging from 0.2 to 0.5 μg/kg/h)	After 1 h the dexmedetomidine was given, the patient was no longer agitated, with a Ramsey Score of three her respiratory rate returned to normal (19 breaths per minute) and SpO2 increased to 97%	Effectiveness of BiPAP was optimized by using dexmedetomidine, with reduced risk of respiratory complications	The patient did not experience bradycardia or hypotension
Duan (48)	Case report	16-year-old primigravida woman	Atypical pneumonia	BPAP and dexmedetomidine (0.2–1.4 g/kg/h)	Dexmedetomidine help in BPAP tolerance du reducing to anxiety and agitation. Improvement of respiratory distress and hypoxemia	Dex allow the patient to tolerate the application of BiPAP	Not reported
Stockton (50)	Case report	58-year old woman	Shortness of breath associated with COVID-19	HFNC with non-rebreather mask Dexmedetomidine infusion	Saturation increased and she was more comfortable	Dex decreased agitation allowing the patient to tolerate better HFNC	Bradycardia, ma Its side effect of bradycardia appears to be well tolerated
Takasaki (49)	2 case reports	Case 1: 65 year old man Case 2: 32 year old woman	Case 1 with severe asthma attack Case 2 with exacerbated asthma,	Case 1: NIV dexmedetomidine for agitation IV (loading dose 3 μg·kg^−1^·min^−1^, followed by a continuous infusion at 0.2–0.6 μg·kg^−1^·min^−1^) Case 2: NIV Dexmedetomidine IV initial 3 μg·kg^−1^·min^−1^ for 10 min for initial loading, then by continuous infusion at 0.2–0.6 μg·kg^−1^·min^−1^.	Case 1: respiratory symptoms improved. Case 2: paradoxical respiratory movement disappeared, gas exchange improved	Dexmedetomidine was effective and safe for sedation during NPPV in two asthmatic patient with acute respiratory failure	Case 1 required dopamine infusion for hypotension

ARF, Acute Respiratory Failure; ARDS, Acute Respiratory Distress Syndrome; BIS, Bispectral Index; COPD, Chronic Obstructive Pulmonary Disease; COVID-19, Coronavirus Disease 2019; CPR, Cardiopulmonary Resuscitation; DEXH, High-dose Dexmedetomidine; DEXL, Low-dose Dexmedetomidine; DNI, Do Not Intubate; ECMO, Extracorporeal Membrane Oxygenation; ED, Emergency Department; HAE, Hemodynamic Adverse Events; HFNC, High-Flow Nasal Cannula; CU, Intensive Care Unit; IMV, Invasive Mechanical Ventilation; IQR, Interquartile Range; IV, Intravenous; LOS, Length of Stay; NIMV, Non-Invasive Mechanical Ventilation; NIV, Non-Invasive Ventilation; NPPV, Non-Invasive Positive Pressure Ventilation; PROP, Propofol; RASS, Richmond Agitation-Sedation Scale; RCT, Randomized Controlled Trial; REML, Low-dose Remifentanil; REMH, High-dose Remifentanil; RSS, Ramsay Sedation Scale; SD, Standard Deviation.

### 3.1 Synthesis of the studies

#### 3.1.1 Pediatric studies

Ten studies were included regarding pediatric patients (6, 9, 19–26). No RCT was found; the pediatric evidence base consists entirely of observational studies (one prospective and 5 retrospective), two case series and two case reports, preventing any quantitative synthesis. Furthermore, considering the broad variation of respiratory conditions included in the pediatric studies, a subgroup analysis was not feasible. All the evaluated studies presented low risk of bias in the majority of the items considered (JBI tools for case series and observational studies were used accordingly, Supplementary Figure 1). Consistent variations were observed across studies in terms of drugs used (other than dexmedetomidine), doses, conditions treated, and study designs, rendering it impossible to conduct a meta-analysis of the retrieved articles.

Venkatraman et al. (24) observed that over 80% of children in ICU undergoing NRS for status asthmaticus or bronchiolitis achieve the desired sedation target level, and 2.5% of the patients required endotracheal intubation. Bermudez-Berrezueta et al. (19) reported that sedation with agents including dexmedetomidine can enhance comfort and acceptance in children under 5 years with acute respiratory failure requiring NIV, leading to better respiratory outcomes. However, they did not find significant differences in the rate of NIV failure, endotracheal intubation, or ICU LOS between sedated and non-sedated children. Notably, the authors did not differentiate between dexmedetomidine and other agents, such as midazolam, making it impossible to assess the specific impact of dexmedetomidine accurately. Eidman et al. (6) retrospectively investigated the use of dexmedetomidine in patients under 5 years old with asthma, bronchiolitis, or pneumonia. No significant difference in intubation rates was observed between the sedated and non-sedated patients. However, a trend toward prolonged NIV duration before intubation was noted in those treated with dexmedetomidine. Additionally, the use of dexmedetomidine was associated with an adequate level of sedation and reduced agitation, especially in younger infants. Piastra et al. (22) observed that dexmedetomidine improved comfort, reduced agitation, and favored NIV acceptance, with no patient experiencing NIV discontinuation due to intolerance.

Shutes et al. (23) explored the effects of prolonged dexmedetomidine infusion (up to 72 h) at a median dose of 1 μg/kg/h for up to 45 h. The hemodynamic effects of dexmedetomidine were predictable, resulting in a decrease in heart rate, and, rarely, mild hypotension. Both these cardiorespiratory events were mild and reversible with adjustments such as dose reduction or fluid administration (24). While bradycardia is a known side effect of dexmedetomidine, there are rare reports of episodes of sinus pauses or asystole, which are more common in the adult population. This warrants caution when administering this drug to patients who are also receiving negative chronotropic medications, have a history of cardiac conduction abnormalities or increased vagal tone (21). Paroxysmal supraventricular tachycardia is a rare adverse event of dexmedetomidine which appears to affect not only patients with underlying heart disease. This highlights the importance of monitoring other risk factors associated with arrhythmia development that may require medical intervention, such as abrupt dexmedetomidine withdrawal, concomitant heart drug use, pain, and fever (27).

Only one study found delirium as an additional adverse effect of dexmedetomidine use during high-flow nasal cannula (HFNC) or bilevel positive airway pressure (BiPAP) (20).

The pharmacokinetics of dexmedetomidine, as well as its manageability when administered intranasally, make it a promising tool in the treatment of severe asthma exacerbation in children, especially in an emergency setting. The management of this condition may include sedation to help younger children tolerate respiratory support and prevent progression to respiratory failure. However, the intranasal application has been reported only in very limited cases (9).

### 3.2 Adult studies

Twenty-four studies were conducted on adult patients, nine RCT (8, 28–35), nine observational studies (36–45), and two case series and four case reports (46–50). Different populations of patients were described including those with acute asthma, acute respiratory failure in chronic obstructive pulmonary disease (COPD), acute hypoxemic cardiogenic pulmonary edema and postoperative respiratory failure, acute respiratory distress, dyspnea, COVID-19, blunt chest trauma, pneumonia, congestive heart failure, and patients undergoing cardiac surgery.

All the evaluated studies presented low risk of bias in the majority of the items considered (JBI’s tools for RCTs, observational studies and case series and case reports were used accordingly; Supplementary Figure 2).

#### 3.2.1 Meta-analysis of the adult studies

Following full-text evaluation, seven RCT studies reported data on the main selected outcomes and were included in the meta-analysis (8, 28–30, 33–35). The limited number of studies included, precluded the conduct of subgroup analysis based on the patients’ conditions.

The primary outcome was the incidence of intubation due to NRS failure, selected to evaluate the effectiveness of dexmedetomidine in improving NRS acceptance.

**Figure 2:**
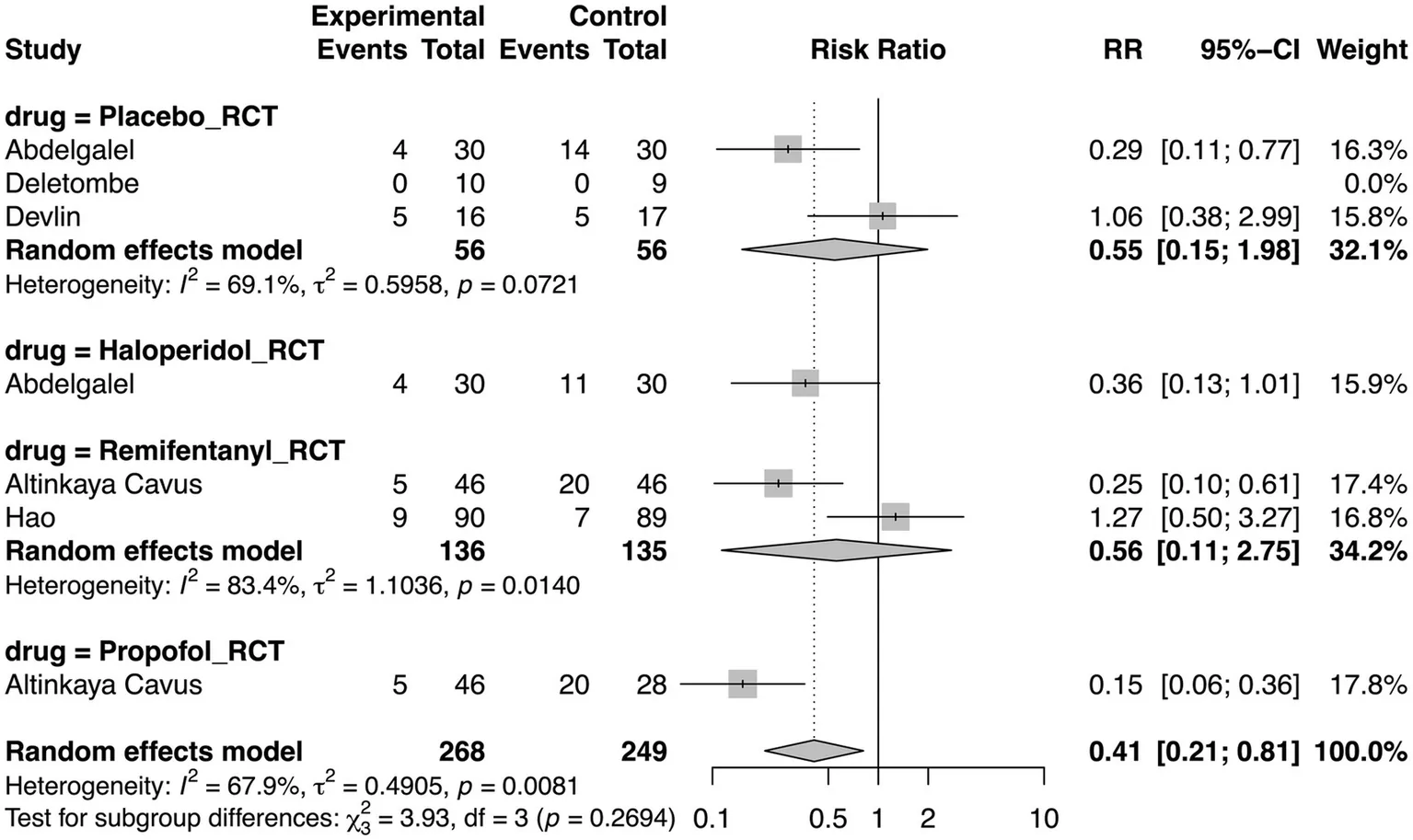
Forest plot of the meta-analysis with intubation rate as the outcome. The effect of dexmedetomidine (“experimental”) on intubation rate (“events”) compared to other drugs or placebo (“control”) was showed. The results from random effect model (REM) were presented, using risk ratio (RR), and 95% confidence interval (CI). Heterogenicity *I*^2^ and *τ*^2^ were also reported. RCT = randomized controlled trial.

In the studies, the use of dexmedetomidine was associated with a lower intubation rate than other pharmacological agents or a placebo. Compared with placebo however, it showed only a trend toward greater efficacy. Heterogeneity was moderate (Figure 2). Influence analysis identified the studies by Altinkaya Cavus et al. (33) (propofol treatment), Devlin et al. (8), and Hao et al. (30) as potential influential outliers, but sensitivity analysis excluding these studies did not alter the overall results of the meta-analysis (Supplementary Figures 3, 4).

Secondary outcomes included mortality rate, delirium incidence, NRS duration, and ICU LOS.

**Figure 3:**
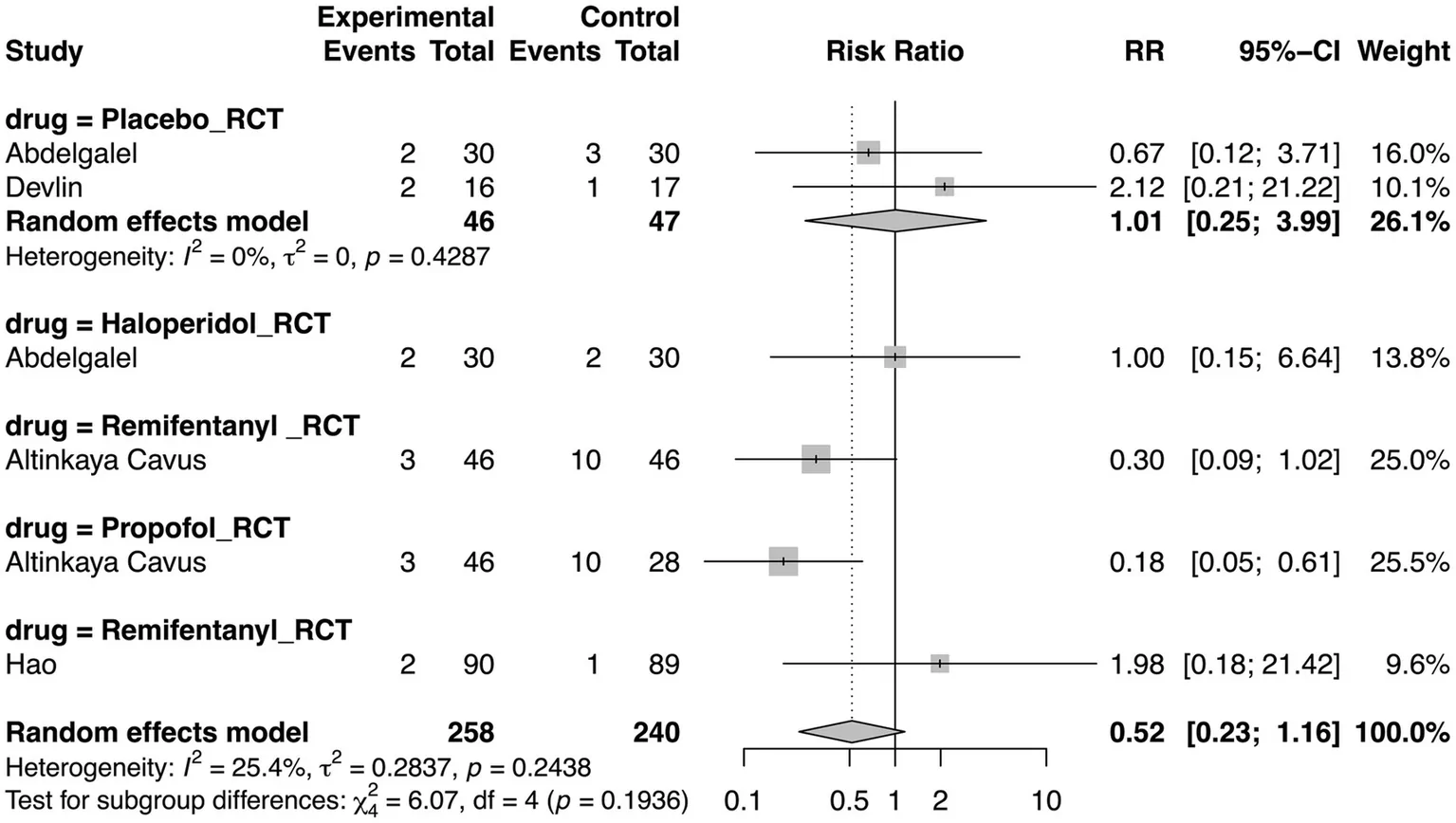
Forest plot of the meta-analysis with mortality rate as outcome. The effect of dexmedetomidine (“experimental”) on mortality rate (“events”) compared to other drugs or placebo (“control”) was showed. The results from random effect model (REM) were presented, using risk ratio (RR), and 95% confidence interval (CI). Heterogenicity *I*^2^ and *τ*^2^ were also reported. RCT = randomized controlled trial.

Treatment with dexmedetomidine had a comparable impact on mortality compared with other treatments. Heterogeneity was low (Figure 3). Although the study by Altinkaya Cavus et al. (33) (propofol treatment) affected the influence diagnostics, the analysis excluding it did not change the overall results (Supplementary Figures 5, 6).

**Figure 4:**
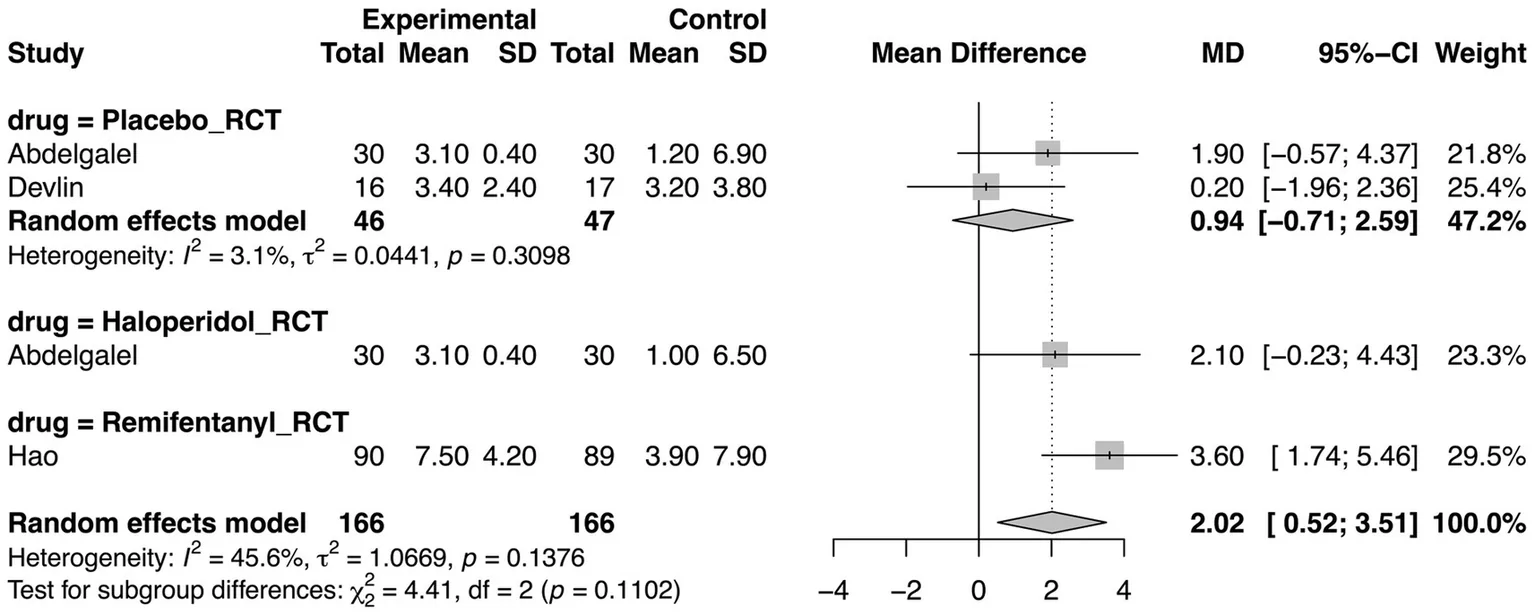
Forest plot of the analysis, outcome noninvasive ventilation duration in hours. The effect of dexmedetomidine (“experimental”) on noninvasive ventilation duration (expressed in mean hours ± standard deviation SD) compared to other drugs or placebo (“control”) were showed. The results from random effect model (REM) were presented, using mean difference (“MD”), and 95% confidence interval (CI). Heterogenicity *I*^2^ and *τ*^2^ were also reported. RCT = randomized controlled trial.

The use of dexmedetomidine showed no significant effect on the duration of NRS compared with other drugs or placebo. However, high heterogeneity may affect the analysis (Figure 4). Influence analysis confirmed this variability (Supplementary Figures 7, 8).

**Figure 5:**
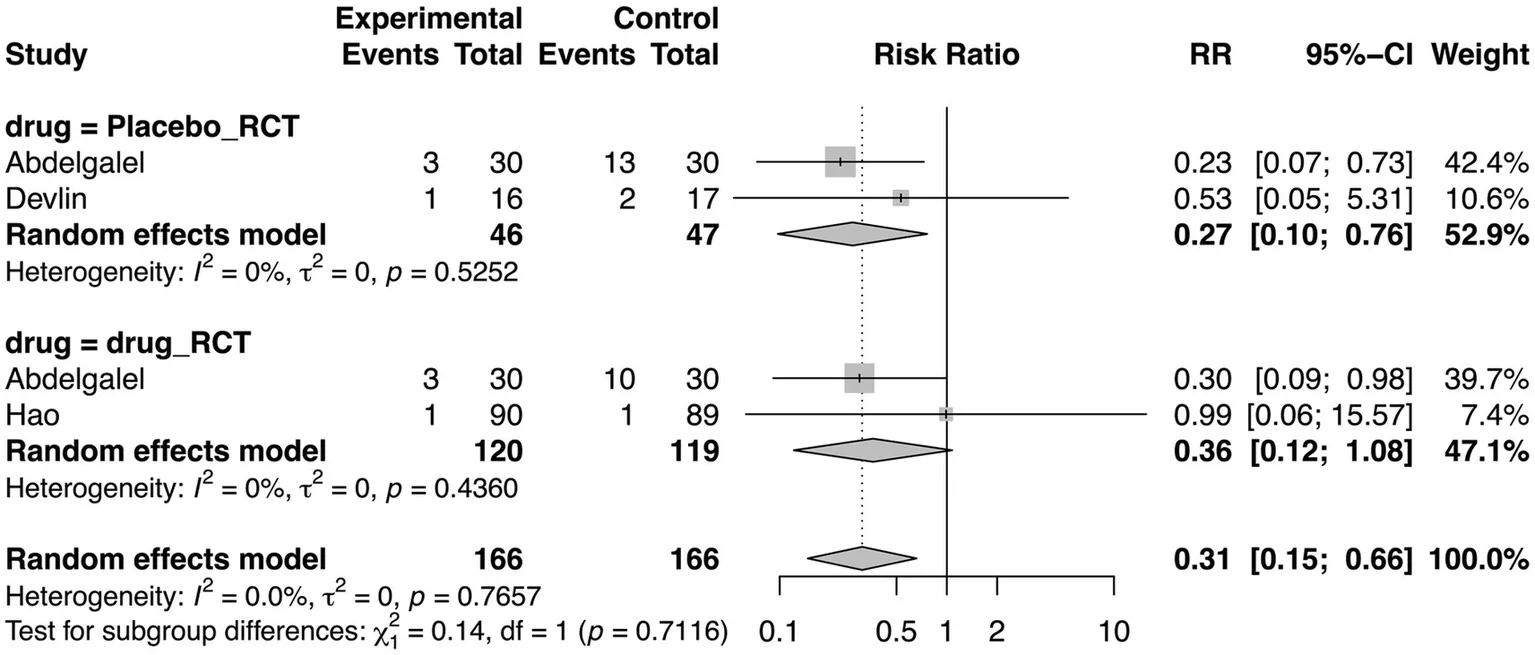
Forest plot of the analysis, outcome length of stay in ICU in days. The effect of dexmedetomidine (“experimental”) on length of stay (LOS) (“events”) (expressed in mean days ± standard deviation SD) respect to other drugs or placebo (“control”) were showed. The results from random effect model (REM) were presented, using mean difference (“MD”), and 95% confidence interval (CI). Heterogenicity *I*^2^ and *τ*^2^ were also reported. RCT = randomized controlled trial.

Patients treated with dexmedetomidine had a longer ICU LOS compared to those receiving other drugs or a placebo. Heterogeneity was moderate (Figure 5), with the study by Devlin et al. (8) and Hao et al. (30) influencing the results (Supplementary Figure 9).

**Figure 6:**
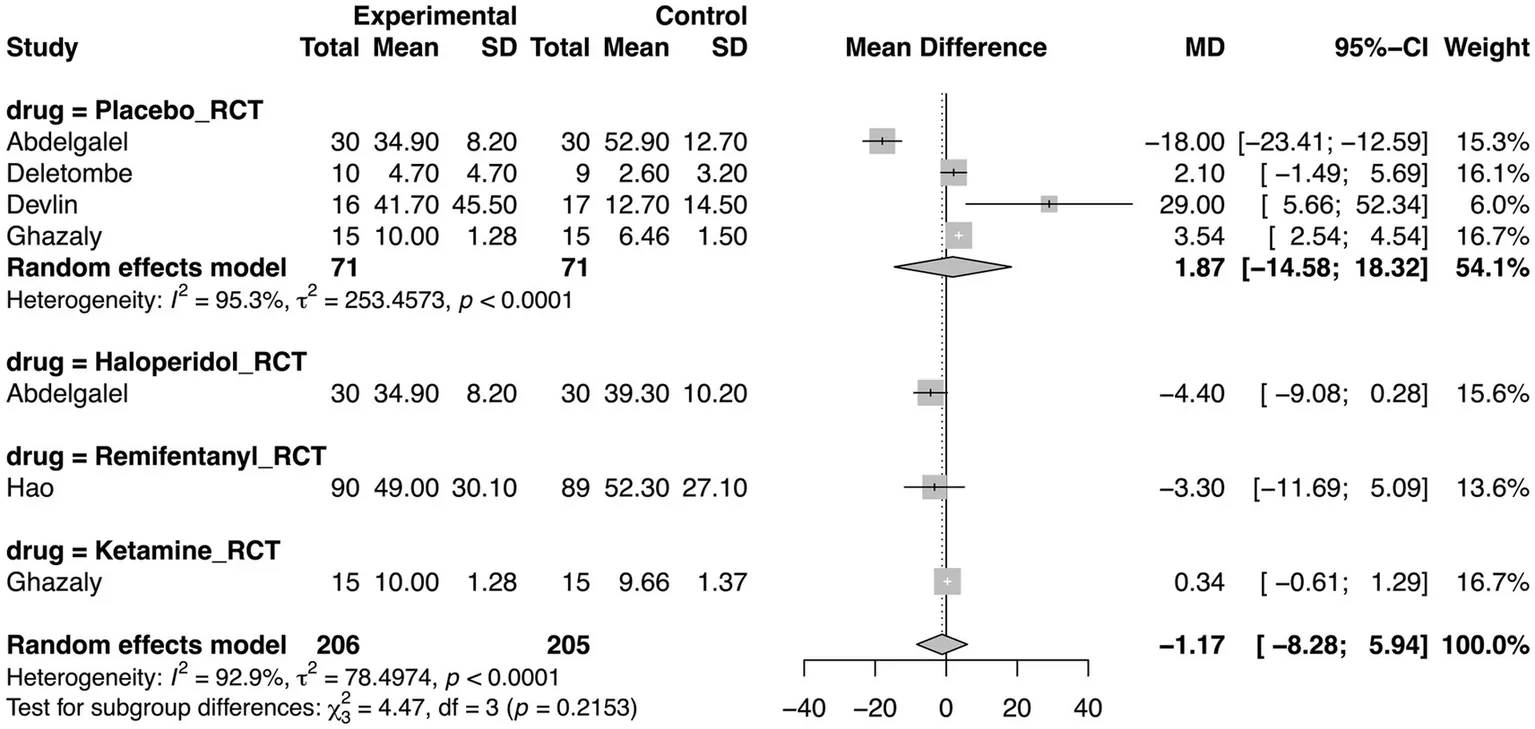
Forest plot of the analysis, outcome delirium incidence. The effect of dexmedetomidine (“experimental”) on delirium incidence (“events”) compared to other drugs or placebo (“control”) was showed. The results from random effect model (REM) were presented, using risk ratio (RR), and 95% confidence interval (CI). Heterogenicity *I*^2^ and *τ*^2^ were also reported. RCT = randomized controlled trial.

Finally, the incidence of delirium was significantly lower in the patients treated with dexmedetomidine compared with those treated with other drugs or a placebo. Heterogeneity was negligible (Figure 6), and no studies were identified as influential (Supplementary Figure 10).

A secondary sensitivity analysis was conducted, including three observational studies (34, 37, 43). Detailed results were reported in Supplementary Figures 11–15. In this analysis, the use of dexmedetomidine was associated with a lower intubation rate and reduced incidence of delirium, as well as a longer ICU LOS, with no significant effect on NRS duration. A reduction in mortality was also observed in the patients treated with dexmedetomidine. However, this finding was not confirmed when the analysis was restricted to RCTs. Therefore, the effect on mortality should be interpreted with caution, as it may reflect residual confounding inherent to observational studies.

The certainty of evidence for each outcome, assessed using the GRADE framework, was summarized in Supplementary Table 2. Certainty was rated as very low for NRS duration, low for intubation, mortality, and ICU LOS, and moderate for delirium incidence.

Due to the lack of individual patient-level data, disaggregated analyses by underlying pathology, age, sex, or gender were not feasible.

## 4 Discussion

This systematic review aimed to investigate the effectiveness of dexmedetomidine in improving NRS tolerance in both pediatric and adult patients with severe acute respiratory distress.

NRS acceptance may be influenced by several factors, including pain, agitation, discomfort, stress, and delirium, which, when present, can negatively affect treatment and often lead to NRS failure.

For children (6, 9, 19–24, 27), the scarcity and heterogeneity of studies investigating the effectiveness of dexmedetomidine for improving NRS acceptance limited the analysis to a descriptive overview of current regimens, and a meta-analysis could not be performed. The available data suggested a potential benefit of dexmedetomidine in facilitating NRS acceptance and achieving adequate sedation; however, these studies also reported potential hemodynamic side effects, preventing a firm conclusion about its safety and efficacy. Additionally, the pediatric studies by Ventrakaman et al. (24), Bermudez-Berrezueta et al. (19), Eidman et al. (6), and Piastra et al. (22) focused on very young patients (under 5 years old), a group in which dexmedetomidine may substantially improve the acceptance of NRS, by reducing discomfort, distress and agitation.

Nevertheless, current evidence does not demonstrate a significant reduction in intubation rates associated with its use. Notably, seven studies were retrospective, only one was prospective, and two were case series (6, 9, 19–26). In addition, one study did not differentiate between sedation agents used (19).

The use of NRS can be particularly challenging in younger children who may feel overwhelmed by clinical situations and hospital procedures. Moreover, children with neurological impairment and/or intellectual disability may be more affected by unfamiliar environments such as hospital settings and often more prone to agitation, which can further exacerbate respiratory compromise. Therefore, the use of safe medication that enhances patient comfort and supports improvement of clinical outcomes is warranted.

Dexmedetomidine, characterized by its rapid onset, easily adjustable sedation level, and minimal effects on respiratory depression, cough reflex, and expectoration, presents several favorable characteristics for use in this setting (6, 9, 19–24, 27).

The evidence for adult patients benefits from the availability of RCTs. Overall, the literature on the effectiveness and safety of dexmedetomidine in the setting of acute severe respiratory distress suggests that it may effectively reduce agitation and facilitate NRS acceptance (8, 28–30, 33, 34, 37, 43).

Based on the meta-analysis results, the use of dexmedetomidine in adults was associated with a lower incidence of intubation and a reduced incidence of delirium compared with other analgesic drugs or placebo. In contrast, the ICU LOS was longer in patients treated with dexmedetomidine, while the duration of NRS and mortality did not differ between those receiving dexmedetomidine, placebo or alternative agents. However, high heterogeneity in patient populations (including acute reparatory failure, acute hypoxemic cardiogenic pulmonary oedema, respiratory distress, post-operative respiratory failure, chronic obstructive pulmonary disease, chest trauma), clinical settings and in statistical estimates calculation may limit the overall robustness of these findings, resulting in low to moderate certainty of evidence.

These results suggested that analgosedation may help improve NRS tolerance in patients with respiratory conditions, potentially supporting the management of the underlying pathology.

Analysis of the studies involving adult patients indicated that the most frequently reported adverse effects associated with dexmedetomidine administration were hemodynamic, with a high prevalence of bradycardia and hypotension. The incidence of these events varied significantly across the studies. Bradycardia was reported in 30% of patients in the study by Simioli et al. (43), compared with 18% in the control group. Other studies report rates of 17.5% (44), 13% (38), 18.2% (34), and 10% (45). The incidence of hypotension showed similar variability. In the retrospective study by Sinnot et al. (44), hypotension was recorded in 39% of patients treated in the emergency department. Other studies reported lower rates, including 17% in the Baumgartner et al. study (38), 8.3% in the Yildirim et al. study (45), and 12.1% Huang et al. study (34). However, it is important to emphasize the clinical significance of these events. Several studies reported that, although common, these adverse effects often did not require substantial therapeutic intervention (34, 44, 45, 50). The study by Baumgartner et al. (38), further elucidated this point, noting that while the overall incidence of any hemodynamic adverse event was 29%, only 12% were considered clinically significant. In their cohort, only 4% of patients required fluid boluses and another 4% required initiation of vasopressors. Other adverse effects, such as vomiting, acute kidney injury, liver injury, shock, tachycardia, and skin erythema, were reported less consistently and at lower frequencies compared to hemodynamic disturbances (28, 34).

Several challenges emerged when comparing results from adult and pediatric studies. The pediatric evidence consists mainly of observational studies without randomized trials, preventing quantitative synthesis, therefore, direct comparisons with adult findings were not possible and should be considered strictly exploratory. While the meta-analysis indicated an association between dexmedetomidine use and a reduced intubation rate in adults, no pooled analyses could be performed for the pediatric population, in the pediatric studies where these data were reported, no significant difference in NRS failure was observed between sedated and non-sedated patients; however, these findings were based on individual study observations rather than aggregated analyses. Indeed, it is important to note that most pediatric studies included in the systematic review were retrospective chart reviews, which precluded the feasibility of performing a meta-analysis, whereas the adult studies were primarily RCTs or observational comparison studies. In these adult investigations, patients were prospectively enrolled according to predefined inclusion and exclusion criteria and treatment doses were standardized. These methodological differences may explain the discrepancies observed between pediatric and adult outcomes.

### 4.1 Limits

This study has several limitations. Although the primary aim was to compare the use of dexmedetomidine in pediatric and adult settings, the pediatric evidence base consists mainly of observational studies without randomized trials, preventing quantitative synthesis. This also limited the feasibility of performing a robust comparative statistical analysis. Among the adult studies, comparisons were restricted to those reporting the outcomes selected for the meta-analysis. Moreover, heterogeneity in clinical settings and the patients population may have influenced the overall findings. Consequently, a definitive statistical comparison between pediatric and adult populations was not feasible, and direct comparisons with adult results should therefore be interpreted cautiously and framed as exploratory. Furthermore, another important limitation of this study is the substantial heterogeneity in both the study populations and the respiratory conditions in both pediatric and adult studies, which limited the ability to draw firm conclusions.

### 4.2 Recommendations and conclusion

Although the limited data prevented us from making definitive recommendations for pediatric patients, dexmedetomidine may be considered to improve NRS acceptance, reduce agitation, and support respiratory recovery (6, 9, 19–24, 27). In adults, dexmedetomidine may be a reasonable sedation option, as previously highlighted by Karim et al. (7) and Yang et al. (51).

Further data are needed on the intranasal route of administration in children, which is often more acceptable and better tolerated than the intravenous route and may be a more practical choice in non-invasive ventilation settings (52, 53).

This study emphasizes the value of dexmedetomidine for sedation in children receiving NIV. Additionally, it underscores the urgent need for well-designed randomized control studies, particularly in the pediatric population, to clearly define the efficacy and optimal dosing of this drug for sedation across various clinical settings. High quality, age-specific studies are indeed essential to address current evidence gaps, support standardized guidelines development and promote safer and more effective use of dexmedetomidine in children.

## Data Availability

The original contributions presented in the study are included in the article/Supplementary material, further inquiries can be directed to the corresponding author.

## Footnotes

1
https://pubmed.ncbi.nlm.nih.gov

2
https://www.webofscience.com

3
www.embase.com

## References

[1] Fu X Wang L Wang G Liu X Wang X Ma S . Delirium in elderly patients with COPD combined with respiratory failure undergoing mechanical ventilation: a prospective cohort study. BMC Pulm Med. (2022) 22:266. doi: 10.1186/s12890-022-02052-5, 35810306 PMC9271245

[2] Chan K-Y Cheng LSL Mak IWC Ng S-W Yiu MGC Chu C-M. Delirium is a strong predictor of mortality in patients receiving non-invasive positive pressure ventilation. Lung. (2017) 195:115–25. doi: 10.1007/s00408-016-9955-3, 27787611

[3] Cammarota G Simonte R De Robertis E. Comfort during non-invasive ventilation. Front Med. (2022) 9:874250. doi: 10.3389/fmed.2022.874250, 35402465 PMC8988041

[4] Abadesso C Nunes P Silvestre C Matias E Loureiro H Almeida H. Non-invasive ventilation in acute respiratory failure in children. Pediatr Rep. (2012) 4:e16. doi: 10.4081/pr.2012.e16, 22802994 PMC3395974

[5] Rosenberg L Traube C. Sedation strategies in children with pediatric acute respiratory distress syndrome (PARDS). Ann Transl Med. (2019) 7:509. doi: 10.21037/atm.2019.09.16, 31728362 PMC6828786

[6] Eidman DB Clauss CL Kelly SA M Rhieu J McCollum S G Couloures K. Dexmedetomidine for sedation during pediatric noninvasive ventilation. Respir Care. (2022) 67:301–7. doi: 10.4187/respcare.09360, 35078930

[7] Karim HM Šarc I Calandra C Spadaro S Mina B Ciobanu LD . Role of sedation and analgesia during noninvasive ventilation: systematic review of recent evidence and recommendations. Indian J Crit Care Med. (2022) 26:938–48. doi: 10.5005/jp-journals-10071-23950, 36042773 PMC9363803

[8] Devlin JW Al-Qadheeb NS Chi A Roberts RJ Qawi I Garpestad E . Efficacy and safety of early dexmedetomidine during noninvasive ventilation for patients with acute respiratory failure. Chest. (2014) 145:1204–12. doi: 10.1378/chest.13-1448, 24577019

[9] Cozzi G Lega S Giorgi R Barbi E. Intranasal dexmedetomidine sedation as adjuvant therapy in acute asthma exacerbation with marked anxiety and agitation. Ann Emerg Med. (2017) 69:125–7. doi: 10.1016/j.annemergmed.2016.08.005, 27776827

[10] Page MJ McKenzie JE Bossuyt PM Boutron I Hoffmann TC Mulrow CD . The PRISMA 2020 statement: an updated guideline for reporting systematic reviews. BMJ. (2021) 372:n71. doi: 10.1136/bmj.n71, 33782057 PMC8005924

[11] Barker TH Stone JC Sears K Klugar M Tufanaru C Leonardi-Bee J . The revised JBI critical appraisal tool for the assessment of risk of bias for randomized controlled trials. JBI Evid Synth. (2023) 21:494–506. doi: 10.11124/JBIES-22-00430, 36727247

[12] Moola S Munn Z Tufanaru C Aromataris E Sears K Sfetcu R . "7. Systematic reviews of etiology and risk". In: Aromataris E Lockwood C Porritt K Pilla B Jordan Z, editors. JBI Manual for Evidence Synthesis (2024)

[13] Munn Z Barker TH Moola S Tufanaru C Stern C McArthur A . Methodological quality of case series studies: an introduction to the JBI critical appraisal tool. JBI Database System Rev Implement Rep. (2019). doi: 10.11124/JBISRIR-D-19-00099 33038125

[14] McGuinness LA Higgins JPT. Risk-of-bias VISualization (robvis): an R package and shiny web app for visualizing risk-of-bias assessments. Res Synth Methods. (2021) 12:55–61. doi: 10.1002/jrsm.1411, 32336025

[15] Rstudio. Rstudio: IDE integrated development environment for R (2023). Available online at: http://www.rstudio.com/ (Accessed June, 1, 2025).

[16] R core Team. R: a language and environment for statistical computing (2023). Available online at: http://www.R-project.org (Accessed June, 1, 2025).

[17] Balduzzi S Rücker G Schwarzer G. How to perform a meta-analysis with R: a practical tutorial. Evid Based Mental Health. (2019) 22:153–60. doi: 10.1136/ebmental-2019-300117, 31563865 PMC10231495

[18] The GRADE Working Group. GRADE handbook for grading quality of evidence and strength of recommendations (2013). Available online at: guidelinedevelopment.org/handbook (Accessed September 1, 2025).

[19] Bermúdez-Barrezueta L Mayordomo-Colunga J Miñambres-Rodríguez M Reyes S Valencia-Ramos J Lopez-Fernandez YM . Implications of sedation during the use of noninvasive ventilation in children with acute respiratory failure (SEDANIV study). Crit Care. (2024) 28:235. doi: 10.1186/s13054-024-04976-2, 38992698 PMC11241858

[20] Christian CE Kim SS Tobias JD. Delirium in pediatric patients with respiratory insufficiency requiring noninvasive ventilation. J Clin Med Res. (2022) 14:357–63. doi: 10.14740/jocmr4805, 36258841 PMC9534187

[21] Lichtsinn K Sehgal I Wilson A. Asystole in 2 pediatric patients during dexmedetomidine infusion. J Pharm Pract. (2023) 36:176–9. doi: 10.1177/08971900211027133, 34165021

[22] Piastra M Pizza A Gaddi S Luca E Genovese O Picconi E . Dexmedetomidine is effective and safe during NIV in infants and young children with acute respiratory failure. BMC Pediatr. (2018) 18:282. doi: 10.1186/s12887-018-1256-y, 30144795 PMC6109351

[23] Shutes BL Gee SW Sargel CL Fink KA Tobias JD. Dexmedetomidine as single continuous sedative during noninvasive ventilation: typical usage, hemodynamic effects, and withdrawal*. Pediatr Crit Care Med. (2018) 19:287–97. doi: 10.1097/PCC.0000000000001451, 29341985

[24] Venkatraman R Hungerford JL Hall MW Moore-Clingenpeel M Tobias JD. Dexmedetomidine for sedation during noninvasive ventilation in pediatric patients*. Pediatr Crit Care Med. (2017) 18:831–7. doi: 10.1097/PCC.0000000000001226, 28598946

[25] Musolino AM Persia S Supino MC Stoppa F Rotondi Aufiero L Nacca R . Helmet continuous positive airway pressure for acute bronchiolitis respiratory failure in a pediatric ward: is it a replicable experience? Children. (2024) 11:1273. doi: 10.3390/children11111273, 39594847 PMC11592809

[26] Cousin VL Corbisier T Rimensberger PC Polito A Bordessoule A. Total face mask with neurally adjusted ventilatory assist as a rescue therapy in infants with severe bronchiolitis. Eur J Pediatr. (2024) 183:2813–7. doi: 10.1007/s00431-024-05543-1, 38581463 PMC11192665

[27] Flores-González JC Estalella-Mendoza A Lechuga-Sancho AM Hernández-González A Rubio-Quiñones F Rodríguez-Campoy P . Supraventricular tachycardia after withdrawal of prolonged dexmedetomidine infusion in a paediatric patient without heart disease. J Clin Pharm Ther. (2017) 42:653–5. doi: 10.1111/jcpt.12564, 28556301

[28] Abdelgalel EF. Dexmedetomidine versus haloperidol for prevention of delirium during non-invasive mechanical ventilation. Egypt J Anaesth. (2016) 32:473–81. doi: 10.1016/j.egja.2016.05.008

[29] Deletombe B Trouve-Buisson T Godon A Falcon D Giorgis-Allemand L Bouzat P . Dexmedetomidine to facilitate non-invasive ventilation after blunt chest trauma: a randomised, double-blind, crossover, placebo-controlled pilot study. Anaesthesia Crit Care Pain Med. (2019) 38:477–83. doi: 10.1016/j.accpm.2019.06.012, 31319192

[30] Hao G Wu J Yu S Liu K Xue Y Gong Q . Remifentanil vs. dexmedetomidine for cardiac surgery patients with noninvasive ventilation intolerance: a multicenter randomized controlled trial. J Intensive Care. (2024) 12:35. doi: 10.1186/s40560-024-00750-2, 39294818 PMC11409483

[31] Ueno Y Sato K Momota K Sato H Nakano Y Akimoto Y . The quality and quantity of sleep on dexmedetomidine during high-flow nasal cannula oxygen therapy in critically ill patients. J Med Investig. (2022) 69:266–72. doi: 10.2152/jmi.69.266, 36244779

[32] Senoglu N Oksuz H Dogan Z Yildiz H Demirkiran H Ekerbicer H. Sedation during noninvasive mechanical ventilation with dexmedetomidine or midazolam: a randomized, double-blind, prospective study. Curr Ther Res. (2010) 71:141–53. doi: 10.1016/j.curtheres.2010.06.003, 24683260 PMC3967280

[33] Altınkaya Çavuş M Gökbulut Bektaş S Turan S. Comparison of clinical safety and efficacy of dexmedetomidine, remifentanil, and propofol in patients who cannot tolerate non-invasive mechanical ventilation: a prospective, randomized, cohort study. Front Med. (2022) 9:995799. doi: 10.3389/fmed.2022.995799, 36111123 PMC9468549

[34] Huang Z Chen Y Yang Z Liu J. Dexmedetomidine versus midazolam for the sedation of patients with non-invasive ventilation failure. Intern Med. (2012) 51:2299–305. doi: 10.2169/internalmedicine.51.7810, 22975538

[35] Ghazaly HF Elansary MM Mahmoud AA Hasanen MK Hassan MM. Dexmedetomidine versus ketamine in improving tolerance to noninvasive ventilation after blunt chest trauma: a randomized, double-blinded, placebo-controlled trial. J Anaesthesiol Clin Pharmacol. (2024) 40:619–25. doi: 10.4103/joacp.joacp_145_23, 39759057 PMC11694855

[36] Akada S Takeda S Yoshida Y Nakazato K Mori M Hongo T . The efficacy of dexmedetomidine in patients with noninvasive ventilation: a preliminary study. Anesth Analg. (2008) 107:167–70. doi: 10.1213/ane.0b013e3181732dc2, 18635484

[37] Basilim A Eljaaly K Aljuhani O Korayem GB Altebainawi AF Aldhmadi WJ . Evaluation of effectiveness and safety of dexmedetomidine in non-mechanically ventilated COVID-19 critically ill patients: a multicentre cohort study. J Intensive Care Med. (2025) 40:74–84. doi: 10.1177/08850666241268498, 39552466

[38] Baumgartner K Joseph M Lothet E Fuller BM. Dexmedetomidine in the emergency department: a prospective observational cohort study. Acad Emerg Med. (2024) 31:263–72. doi: 10.1111/acem.14842, 38060343

[39] Dunbar PJ Peterson R McGrath M Pomponio R Kiser TH Ho PM . Analgesia and sedation use during noninvasive ventilation for acute respiratory failure. Crit Care Med. (2024) 52:1043–53. doi: 10.1097/CCM.0000000000006253, 38506571 PMC13107206

[40] Kim T Kim JS Choi EY Chang Y Choi W-I Hwang J-J . Utilization of pain and sedation therapy on noninvasive mechanical ventilation in Korean intensive care units: a multi-center prospective observational study. Acute Crit Care. (2020) 35:255–62. doi: 10.4266/acc.2020.00164, 33161687 PMC7808848

[41] Matsumoto T Tomii K Tachikawa R Otsuka K Nagata K Otsuka K . Role of sedation for agitated patients undergoing noninvasive ventilation: clinical practice in a tertiary referral hospital. BMC Pulm Med. (2015) 15:71. doi: 10.1186/s12890-015-0072-5, 26164393 PMC4499444

[42] Riccardi A Serra S De Iaco F Fabbri A Shiffer D Voza A. Uncovering the benefits of the ketamine–dexmedetomidine combination for procedural sedation during the Italian COVID-19 pandemic. JCM. (2023) 12:3124. doi: 10.3390/jcm12093124, 37176565 PMC10179324

[43] Simioli F Annunziata A Coppola A Imitazione P Mirizzi AI Marotta A . The role of dexmedetomidine in ARDS: an approach to non-intensive care sedation. Front Med. (2023) 10:1224242. doi: 10.3389/fmed.2023.1224242, 37720511 PMC10502206

[44] Sinnott J Holthaus C Ablordeppey E Wessman B Roberts B Fuller B. The use of dexmedetomidine in the emergency department: a cohort study. WestJEM. (2021) 22:1202–9. doi: 10.5811/westjem.2021.4.50917, 34546899 PMC8463063

[45] Yildirim F Karaman I Yıldırım M Karabacak H. Benefits of dexmedetomidine during noninvasive mechanical ventilation in major abdominal surgery patients with postoperative respiratory failure. Front Surg. (2024) 11:1357492. doi: 10.3389/fsurg.2024.1357492, 38800629 PMC11120960

[46] Akhtar MH Haleem S Tauheed N Khan D. Dexmedetomidine as conduit for non-invasive ventilation (NIV) compliance in COVID-19 and chronic obstructive pulmonary disease (COPD) patients in intensive care unit (ICU) setting: case series. Cureus. (2023) 15:e33981. doi: 10.7759/cureus.33981, 36811041 PMC9938913

[47] DeMuro J Mongelli M Hanna A. Use of dexmedetomidine to facilitate non-invasive ventilation. Int J Crit Illn Inj Sci. (2013) 3:274–5. doi: 10.4103/2229-5151.124161, 24459626 PMC3891195

[48] Duan M Lee J Bittner EA. Dexmedetomidine for sedation in the parturient with respiratory failure requiring noninvasive ventilation. Respir Care. (2012) 57:1967–9. doi: 10.4187/respcare.01733, 22709960

[49] Takasaki Y Kido T Semba K. Dexmedetomidine facilitates induction of noninvasive positive pressure ventilation for acute respiratory failure in patients with severe asthma. J Anesth. (2009) 23:147–50. doi: 10.1007/s00540-008-0712-5, 19234843

[50] Stockton J Kyle-Sidell C. Dexmedetomidine and worsening hypoxemia in the setting of COVID-19: a case report. Am J Emerg Med. (2020) 38:2247.e1–2. doi: 10.1016/j.ajem.2020.05.066, 32475761 PMC7251409

[51] Yang B Gao L Tong Z. Sedation and analgesia strategies for non-invasive mechanical ventilation: a systematic review and meta-analysis. Heart Lung. (2024) 63:42–50. doi: 10.1016/j.hrtlng.2023.09.005, 37769542

[52] Marzuillo P Calligaris L Amoroso S Barbi E. Narrative review shows that the short-term use of ketorolac is safe and effective in the management of moderate-to-severe pain in children. Acta Paediatr. (2018) 107:560–7. doi: 10.1111/apa.14189, 29247538

[53] Mahmoud M Barbi E Mason KP. Dexmedetomidine: what’s new for pediatrics? A narrative review. JCM. (2020) 9:2724. doi: 10.3390/jcm9092724, 32846947 PMC7565844

