# Supplementary material for: Dexmedetomidine for acute respiratory conditions requiring non-invasive respiratory support in pediatric and adult patients: systematic review and meta-analysis of the literature
Source: Front Med (Lausanne). 2026 Apr 24;13:1740700. doi: 10.3389/fmed.2026.1740700 (PMC13153069; doi:10.3389/fmed.2026.1740700)

**Table supplementary 1. Detailed Search strategy in the databases**

Last update: 15^th^ September 2025.

| **Database** | **Search string** |
| --- | --- |
| **PubMed** | ((((Non-invasive ventilation) OR (CPAP) OR (high flow cannula) OR (dyspnea) OR (acute respiratory distress) OR ( acute respiratory failure) OR (bronchiolitis) OR(asthma)) AND (dexmedetomidine) ) |
| **Embase** | (('noninvasive ventilation' OR 'continuous positive airway pressure' OR 'high flow cannula' OR 'dyspnea' OR 'acute respiratory distress' OR 'acute respiratory failure' OR 'bronchiolitis' OR 'asthma') AND 'dexmedetomidine') |
| **Web of Science** | ((ALL=noninvasive ventilation) OR (ALL=continuous positive airway pressure) OR (ALL=high flow cannula) OR ( ALL=dyspnea) OR (ALL=acute respiratory distress) OR (ALL=acute respiratory failure) OR (ALL=bronchiolitis) OR (ALL=asthma))AND (ALL=dexmedetomidine) |

**Table supplementary 2.** Summary of findings accordingly to GRADE framework.

Certainty of evidence rated using GRADE (considering risk of bias, inconsistency, indirectness, imprecision, and publication bias)

| **Certainty assessment** | | | | | | | **№ of patients** | | **Effect** | **Certainty** | **Impact** |
| --- | --- | --- | --- | --- | --- | --- | --- | --- | --- | --- | --- |
| **№ of studies** | **Study design** | **Risk of bias** | **Inconsistency** | **Indirectness** | **Imprecision** | **Other considerations** | **Dexmede tomidine** | **Other drugs**  **placebo** | **Risk ratio or MD (95% CI confidence interval)** |  |  |
| **Outcome: rate of intubation in NIV patients treated with dexmedetomidine** | | | | | | | | | | | |
| 5 | randomized controlled trials | not serious | serious | serious | not serious | - | 268 | 249 | REM 0.41  [0.21; 0.81] | ⨁⨁◯◯ Low | The use of dexmedetomidine reduce the risk of intubation |
| **Outcome: rate of mortality in NIV patients treated with dexmedetomidine** | | | | | | | | | | | |
| 4 | randomized controlled trials | not serious | not serious | serious | serious |  | 258 | 240 | REM 0.52  [0.23; 1.16] | ⨁⨁◯◯ Low | The use of dexmedetomidine did not impact on the risk of mortality |
| **Outcome: duration of NIV patients treated with dexmedetomidine** | | | | | | | | | | | |
| 5 | randomized controlled trials | not serious | very serious | serious | serious | - | 206 | 205 | REM -1.17  [-8.28; 5.94] | ⨁◯◯◯ Very Low | Use of dexmedetomidine did not change the length of NRS |
| **Outcome: duration of length of stay in ICU of NIV patients treated with dexmedetomidine** | | | | | | | | | | | |
| 3 | randomized controlled trials | not serious | serious | serious | not serious | - | 230 | 319 | REM 2.02  [0.52; 3.51] | ⨁⨁◯◯ Low | Patients treated with dexmedetomidine incremented length of stay in ICU |
| **Outcome: rates of delirium in NIV patients treated with dexmedetomidine** | | | | | | | | | | | |
| 3 | randomized controlled trials | not serious | not serious | serious | not serious | - | 166 | 166 | REM 0.31  [0.15; 0.66] | ⨁⨁⨁◯ Moderate | The use of dexmedetomidine reduces the incidence of delirium |

ICU= intensive care unit, MD= mean difference, NRS= non invasive respiratory support, REM=random effect model

**Figure Supplementary 1:** Risk of bias assessment for adult studies

**Figure Supplementary 2:** Risk of bias assessment for pediatric studies

Figure supplementary 3. Influence analysis for studies reporting intubation rate as the outcome.

Figure supplementary 4. Forest plot of the analysis with intubation rate as the outcome, excluding the studies by Altinkaya Cavus et al. (2022, propofol treatment), Devlin et al. (2014), and Hao et al. (2024).

The effect of dexmedetomidine (“experimental”) on intubation rate (“events”) compared to other drugs or placebo (“control”) was showed. The results from Random Effect model (REM) were presented, using risk ratio (RR), and 95% confidence interval (CI). Heterogenicity I^2^ and τ^2^ were also reported.

RCT = Randomized Controlled Trial.

Figure supplementary 5. Influence analysis for studies reporting mortality rate as the outcome.

Figure supplementary 6. Forest plot of the meta-analysis with mortality rate as outcome excluding the study by Altınkaya Cavus et al. 2022 (propofol treatment).

The effect of dexmedetomidine (“experimental”) on mortality rate (“events”) compared to other drugs or placebo (“control”) was showed. The results from Random Effect model (REM) were presented, using risk ratio (RR), and 95% confidence interval (CI). Heterogenicity I^2^ and τ^2^ were also reported.

RCT = Randomized Controlled Trial.

Figure supplementary 7. Influence analysis for studies reporting non invasive respiratory support duration (hours) as the outcome.

**Figure supplementary 8**. Forest plot of the analysis, outcome noninvasive ventilation duration in hours excluding the studies by Ghazaly et al and Abdelgalel et al. (placebo treatment).

The effect of dexmedetomidine (“experimental”) on noninvasive ventilation duration (expressed in mean hours ± standard deviation SD) compared to other drugs or placebo (“control”) were showed. The results from Random Effect model (REM) were presented, using mean difference (“MD”), and 95% confidence interval (CI). Heterogenicity I^2^ and τ^2^ were also reported.

RCT = Randomized Controlled Trial

Figure supplementary 9. Influence analysis for studies reporting length of stay (LOS in hours) as the outcome.

Figure supplementary 10. Influence analysis for studies reporting incidence of delirium as the outcome.

Figure supplementary 11. Forest plot of the meta-analysis with intubation rate as the outcome.

Both Randomized Controlled Trial (RCT) and observational studies were included.

The effect of dexmedetomidine (“experimental”) on intubation rate (“events”) compared to other drugs or placebo (“control”) was showed. The results from Random Effect model (REM) were presented, using risk ratio (RR), and 95% confidence interval (CI). Heterogenicity I^2^ and τ^2^ were also reported.

RCT = Randomized Controlled Trial


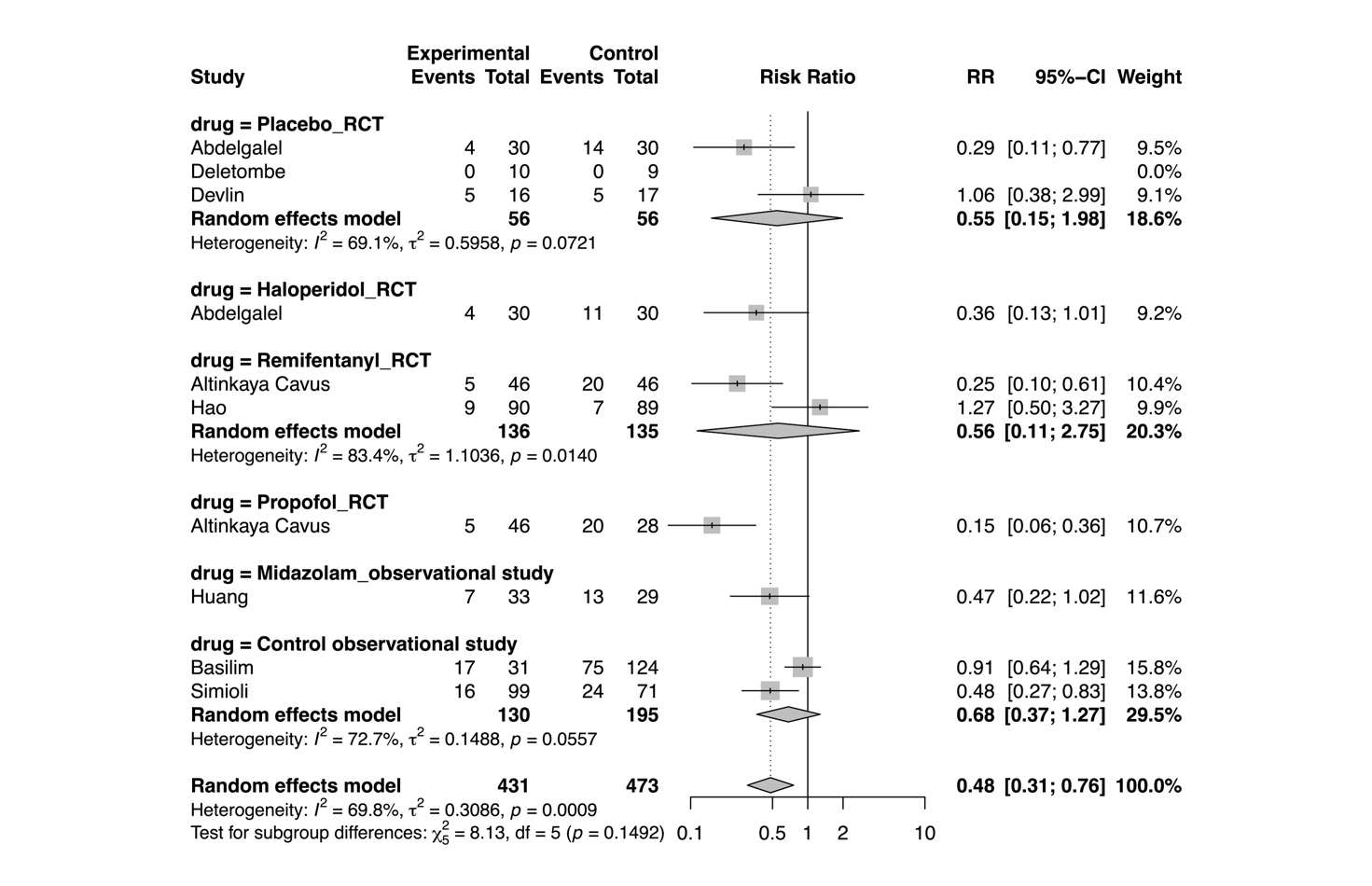


Figure supplementary 12. Forest plot of the meta-analysis with mortality rate as outcome.

Both Randomized Controlled Trial (RCT) and observational studies were included.

The effect of dexmedetomidine (“experimental”) on mortality rate (“events”) compared to other drugs or placebo (“control”) was showed. The results from Random Effect model (REM) were presented, using risk ratio (RR), and 95% confidence interval (CI). Heterogenicity I^2^ and τ^2^ were also reported.

RCT = Randomized Controlled Trial


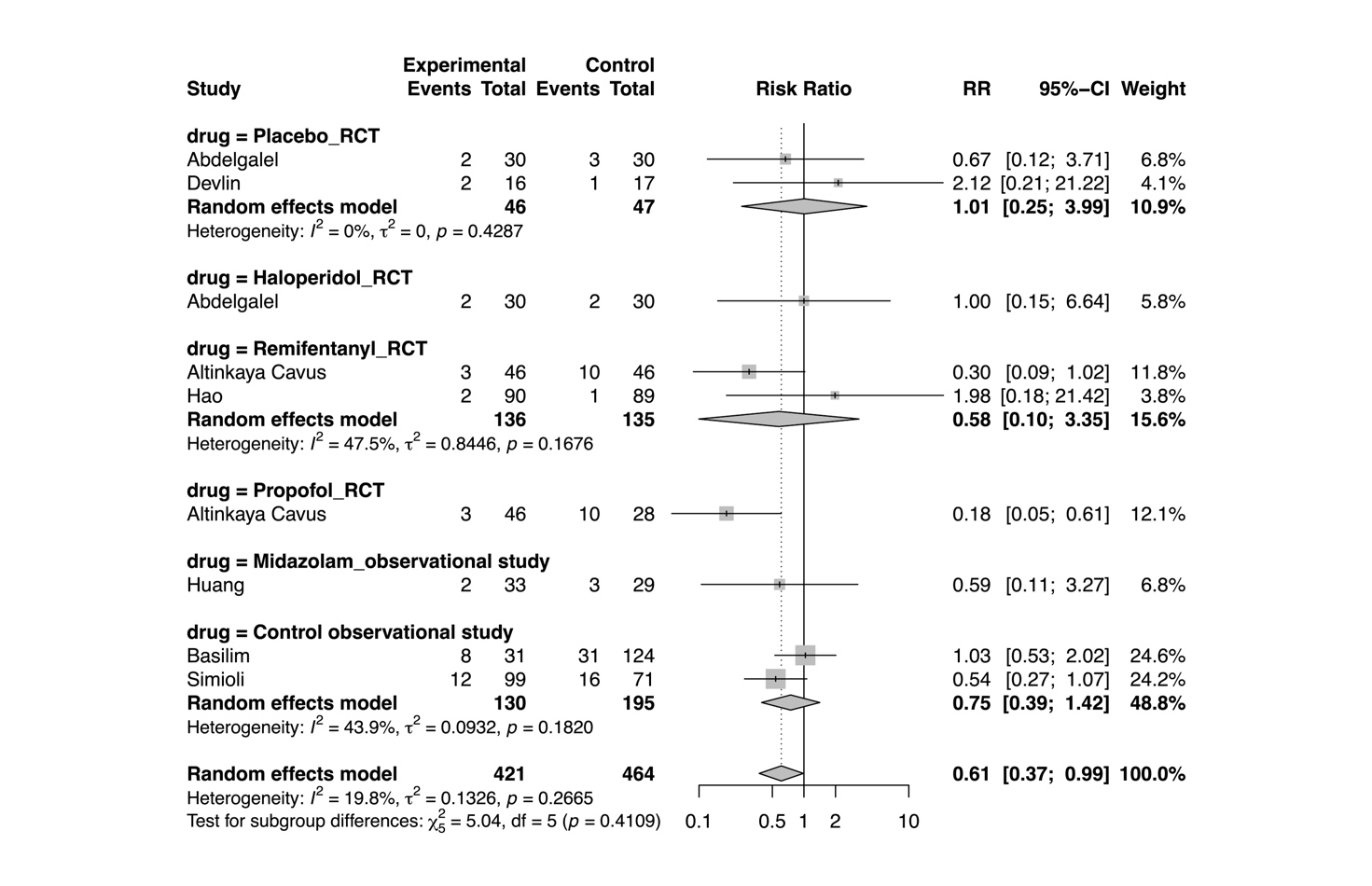


Figure supplementary 13. Forest plot of the analysis, outcome noninvasive respiratory support duration in hours.

Both Randomized Controlled Trial (RCT) and observational studies were included.

The effect of dexmedetomidine (“experimental”) on noninvasive ventilation duration (expressed in mean hours ± standard deviation SD) compared to other drugs or placebo (“control”) were showed. The results from Random Effect model (REM) were presented, using mean difference (“MD”), and 95% confidence interval (CI). Heterogenicity I^2^ and τ^2^ were also reported.

RCT = Randomized Controlled Trial


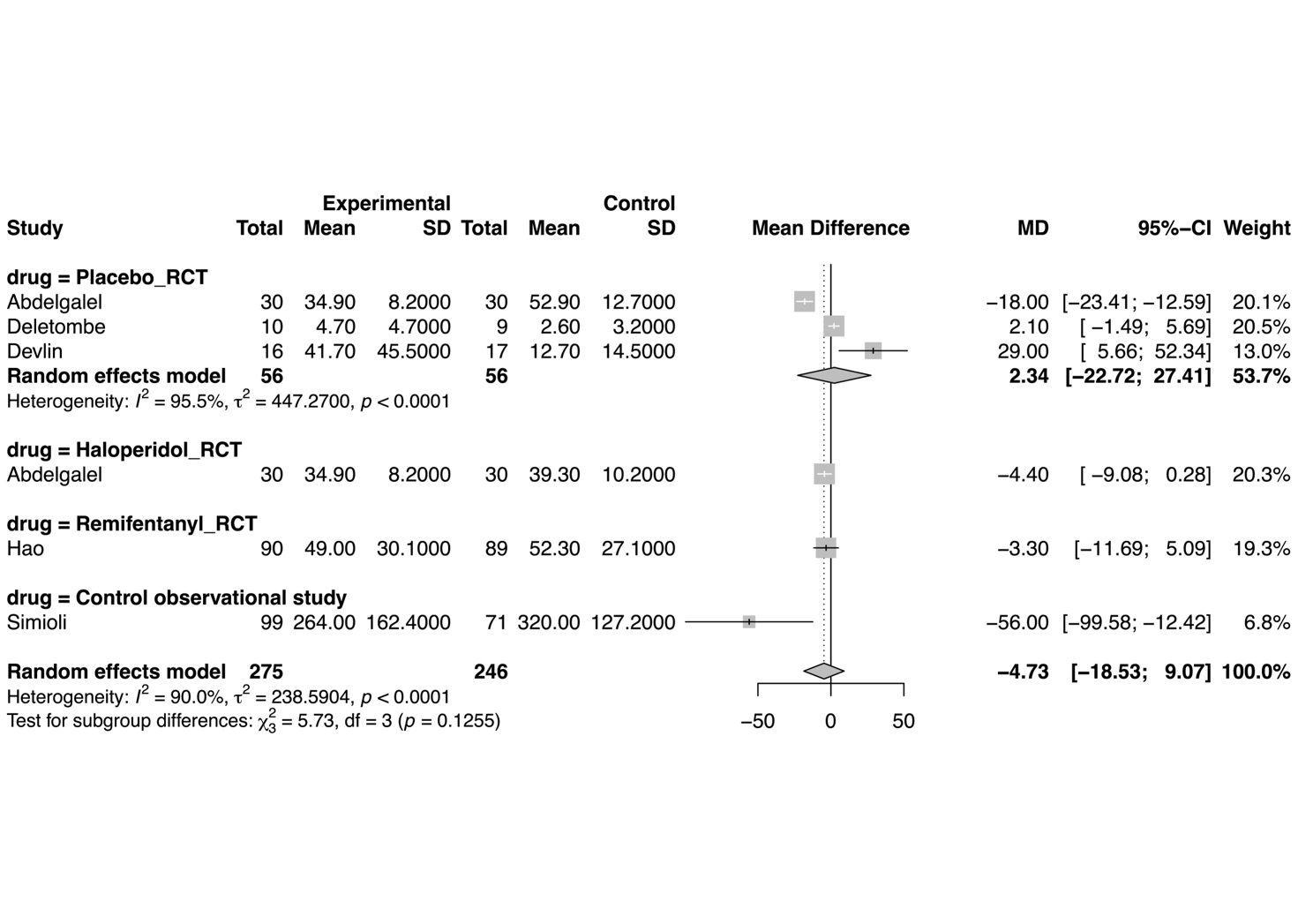


Figure supplementary 14. Forest plot of the analysis, outcome length of stay in intensive care unit in days.

Both Randomized Controlled Trial (RCT) and observational studies were included.

The effect of dexmedetomidine (“experimental”) on length of stay (LOS) (“events”) (expressed in mean days ± standard deviation SD) respect to other drugs or placebo (“control”) were showed. The results from Random Effect model (REM) were presented, using mean difference (“MD”), and 95% confidence interval (CI). Heterogenicity I^2^ and τ^2^ were also reported.

RCT = Randomized Controlled Trial


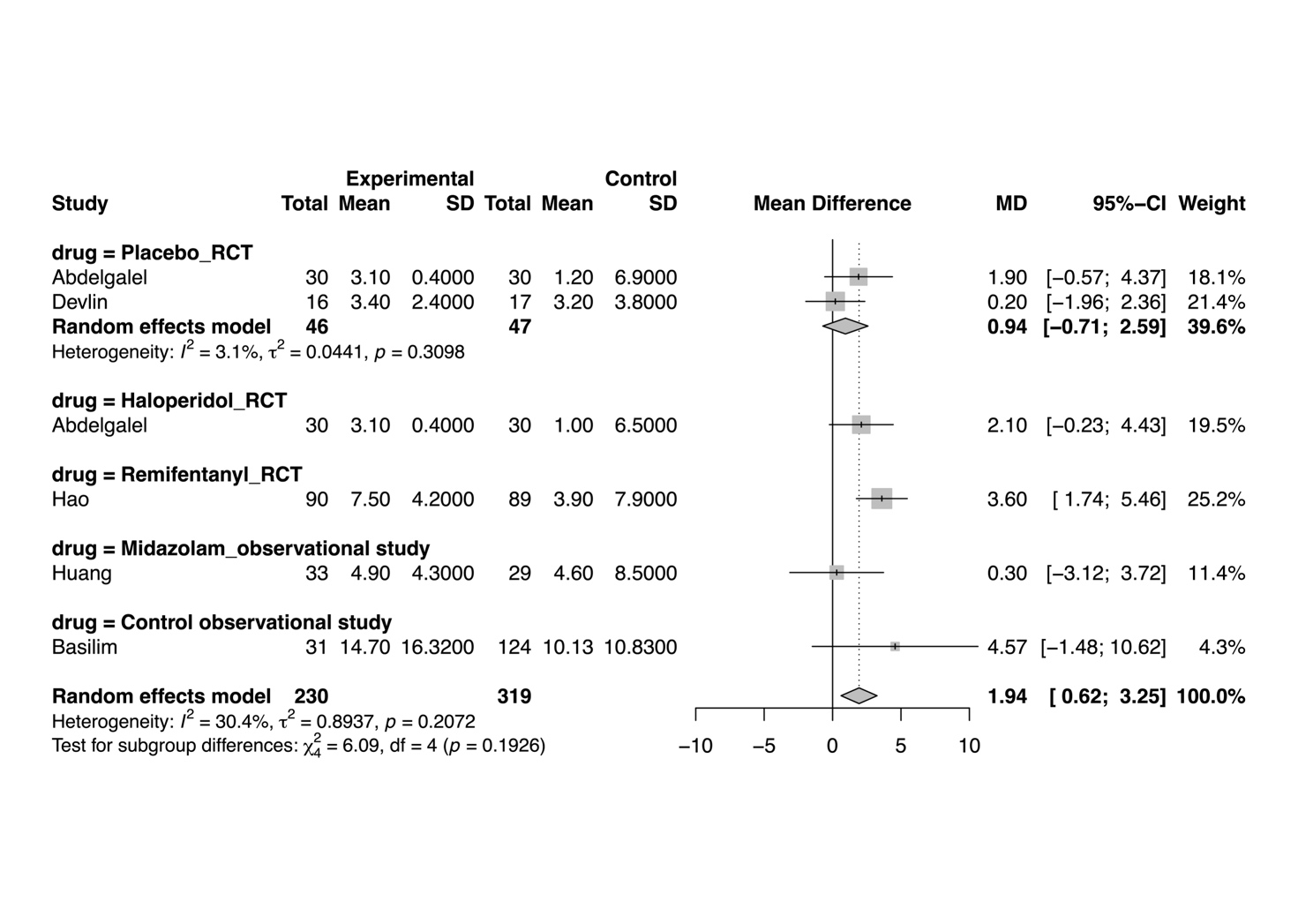


Figure supplementary 15. Forest plot of the analysis, outcome delirium incidence

Both Randomized Controlled Trial (RCT) and observational studies were included.

The effect of dexmedetomidine (“experimental”) on delirium incidence (“events”) compared to other drugs or placebo (“control”) was showed. The results from Random Effect model (REM) were presented, using risk ratio (RR), and 95% confidence interval (CI). Heterogenicity I^2^ and τ^2^ were also reported.

RCT = Randomized Controlled Trial


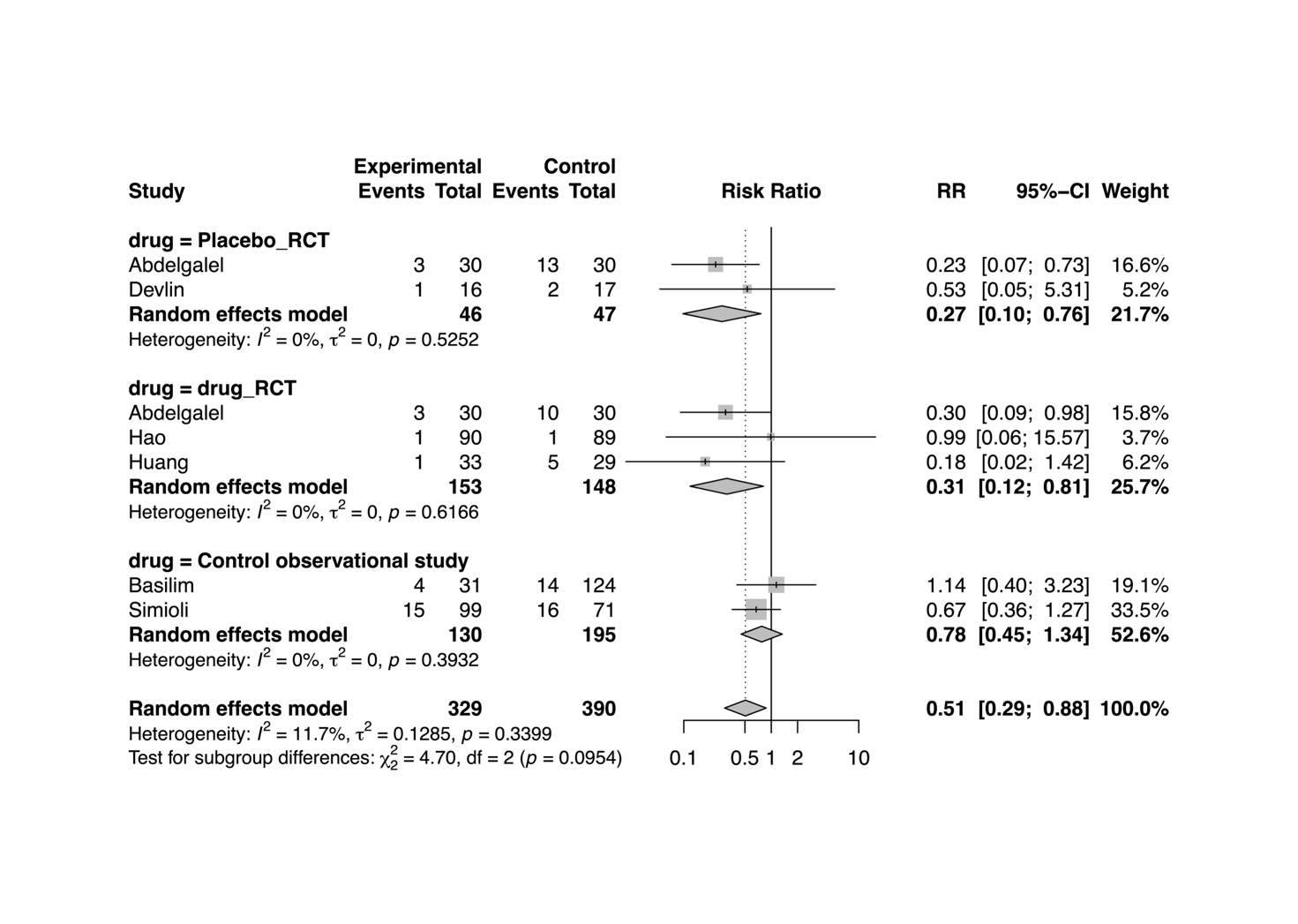

Supplement: Supplementary file 1 [file Supplementary_file_1.docx]
